# Nanoheterostructures (NHS) and Their Applications in Nanomedicine: Focusing on In Vivo Studies

**DOI:** 10.3390/ma12010139

**Published:** 2019-01-03

**Authors:** Alessandra Quarta, Clara Piccirillo, Giacomo Mandriota, Riccardo Di Corato

**Affiliations:** 1 CNR NANOTEC—Institute of Nanotechnology, c/o Campus Ecotekne, University of Salento, Via Monteroni, 73100 Lecce, Italy; clara.piccirillo@nanotec.cnr.it; 2 Department of Mathematics and Physics “E. De Giorgi”, University of Salento, via Arnesano, 73100 Lecce, Italy; giacomo.mandriota@unisalento.it

**Keywords:** nanoheterostructures, biomedicine, imaging, hyperthermia, photothermal therapy, in vivo testing

## Abstract

Inorganic nanoparticles have great potential for application in many fields, including nanomedicine. Within this class of materials, inorganic nanoheterostructures (NHS) look particularly promising as they can be formulated as the combination of different domains; this can lead to nanosystems with different functional properties, which, therefore, can perform different functions at the same time. This review reports on the latest development in the synthesis of advanced NHS for biomedicine and on the tests of their functional properties in in vivo studies. The literature discussed here focuses on the diagnostic and therapeutic applications with special emphasis on cancer. Considering the diagnostics, a description of the NHS for cancer imaging and multimodal imaging is reported; more specifically, NHS for magnetic resonance, computed tomography and luminescence imaging are considered. As for the therapeutics, NHS employed in magnetic hyperthermia or photothermal therapies are reported. Examples of NHS for cancer theranostics are also presented, emphasizing their dual usability in vivo, as imaging and therapeutic tools. Overall, NHS show a great potential for biomedicine application; further studies, however, are necessary regarding the safety associated to their use.

## 1. Introduction

In recent years, nanomedicine has been rapidly emerging as an important branch of nanotechnology, with potential applications in the use of in-vivo nanomaterials. Indeed, the term nanomedicine includes a large variety of disciplines, from materials science to clinical medicine, from electronics to experimental biology [1].

Considering in particular synthetic materials applied to biomedicine, inorganic nanocrystals have been attracting increasing attention due to their specific physiochemical properties; indeed, different formulations have been developed, which lead to a variety of applications, including diagnostics (i.e., contrast agents), therapeutic actuators and nanocarriers (i.e., drug delivery) [2]. Moreover, different properties and functions can be combined into a single nanostructure, to produce multifunctional systems, which can be employed in both diagnosis and therapy, also defined as “theranostics”. Other examples can be the detection of a specific biological target (a specific tissue or a tumor mass) by two different imaging techniques.

Despite these promising properties, nanoparticles have not yet become commonly used in medicine; this is due to different reasons, as summarized below.

Some of the issues associated with the use of nanoparticles in medicine are the same as for nanocrystals in other fields, for instance, the protocols used for their synthesis could be expensive, leading to a high cost per gram of final product. Moreover, often nanoparticles are produced with processes with relatively low yield; this makes it difficult to prepare sizeable amount of material, which could be effectively used for commercial and industrial applications.

Another crucial point is the colloidal stability of the nanoparticles, as this could affect their use over prolonged periods. To address this, efforts have been made to improve the characteristics of the particle coatings; the introduction of new synthetic or natural polymers as stabilizer molecules is considered. For application in medicine, for instance, such coatings could reduce the aggregation of the particles in the bloodstream and the absorption of serum proteins [3].

Considering issues that are more specific for the clinical field, one additional element could be that some nanocrystals may have a hydrophobic nature, which is incompatible with medical application and requires further surface treatment for dispersing particles in biological media [4,5]. Moreover, there is some concern regarding possible side effects from the use of nanomaterials in vivo. Because of this, the study of the toxicity of nanomaterials (nanotoxicity) is of crucial importance. Indeed, regarding this issue, several of its aspects have to be taken into account; the acute toxicity, for instance, is a very important element, and usually is related to the structure of the nanosystems, their chemical composition, their surface coating and the administered dose. The fate of the nanoparticles and their long-term effects on cells and tissues after administration are also of major importance [6,7]. Another essential point is the real efficacy of the nanosystems. Indeed, many studies show the appealing properties of nanoparticles through in vitro tests; when the same systems are tested in animal models, however, their efficacy is seriously compromised [8].

Considering these elements, it is clear why in medicine there is a gap between scientists developing the nanosystems and those using them for clinical applications, and why, in comparison with other fields, such as energy or electronics, this gap is larger. Despite these limitations, however, due to the unique properties of the nanoscale materials, there is still interest from researchers in this field, aimed at improving the quality and the features of these materials and to bridging these gaps. It is worth mentioning that, for instance, advances in nanomaterials displaying novel or implemented properties allows reducing the administered doses and, in turn, to limit the adverse effects due to the accumulation of toxic elements (i.e., heavy metals and reactive ion species) in tissue or organs [9,10,11].

From the medical/biological point of view, there is a particular interest in the development of inorganic nanoheterostrutures (NHS); with these, in fact, there is the possibility to combine different domains and thus multiple properties in a single nanoscale structure. This, in turn, allows: (i) following the fate of a single entity inside the body; (ii) reducing the administered doses and, consequently, mitigate the adverse effects; (iii) co-localizing the signals deriving from different particles and techniques, with increased accuracy; and (iv) combining diagnosis and therapy, exploiting the healing effect of particles. Moreover, NHS offer the possibility to perform a differential functionalization, by anchoring different moieties on the surface of different domains; examples include polymer and nucleotide [12], polymer and peptide [13], antigen and chemotherapy drug [14] or antigen and fluorophore [15]. This can be done by exploiting the different affinity of a material for specific functional groups.

The building blocks of the NHS used in nanomedicine are either magnetic, metallic or luminescent nanoparticles. The first two classes can be employed both in diagnosis and in therapy; for diagnosis, magnetic resonance imaging (MRI) and computed tomography (CT) are the most commonly used, while for therapy the main application is the photothermal effect. Luminescent particles, on the other hand, are exploited in diagnosis by optical imaging.

The focus of this review is to present the heterostructures synthesized through the direct growth/assembly of an inorganic domain on an original seed. This approach generally leads to the preparation of core@shell nanoparticles (dimers or trimers), in which the domains share only a facet of their crystalline structure; even multibranched particles can be prepared. The NHS developed thus far are reported considering the corresponding field of application. More specifically, after a brief description of the main imaging techniques (MRI, CT and luminescence imaging), an analysis of the heterosystems used in diagnosis is considered. Thereafter, the heterostructures with therapeutic or theranostic potential are reported.

## 2. Diagnostic Imaging in Nanomedicine

An accurate diagnosis of diseases and the early identification of biological targets are crucial and highly desirable to decide an effective and decisive treatment. It is therefore reasonable that diagnostic imaging represents the first and probably the most investigated application field for inorganic nanoparticles in nanomedicine. The requirement for innovative contrast agents and the availability of different diagnostic setups led to increasing research in this direction.

Considering the bioimaging techniques, the most employed are those based on magnetic resonance and on optical/luminescent imaging. When imaging is performed in vivo to screen the whole body, other techniques such as X ray-CT and Positron Emission Tomography (PET) can provide additional information about tissues/organs or metabolic alterations. Both spatial accuracy and in-depth resolution can vary greatly depending on the technique. Hence, the choice of the most appropriate technique may depend on several elements, for instance the subject under investigation; for each case, a proper evaluation of benefits and limitations should be considered [16,17].

At present, the most employed nanoparticles for imaging are those showing magnetic behavior, due to the combination of different important properties such as good biocompatibility, extreme versatility and highly diagnostic performance via magnetic resonance. Because of this, a magnetic nanoparticles-based formulation was one of the first nanosystems to be approved by FDA, for MRI detection of hepatic lesion [18].

Research on magnetic nanoparticles aims to achieve structures with advanced characteristics and performances; features such as shape, size, composition, anisotropy and crystalline purity can be optimized to enhance the magnetic properties and obtain the best performing actuator.

In addition, new magnetic heterostructures are synthetized, for advanced medical applications. In few cases, the research focuses on structures with enhanced magnetic properties, the most common examples being the core@shell magnetic particles; in the majority of cases, however, the new structures consist of the combination of different kinds of materials, to obtain nanostructures usable for multiple imaging techniques. Indeed, the co-localization of signals deriving from the combined use of different imaging techniques (i.e., MRI and CT or optical imaging) can provide an advanced diagnosis; improvements can be achieved for both spatial and temporal resolution and intensity of the signals. The possibility to repeat the diagnostic investigation can also be attained.

Considering CT imaging, metal nanoparticles such as gold and silver can play a crucial role. These nanoparticles, in fact, can lead to a significant enhancement of the CT signal; moreover, they also present other advantages, in terms of tissue accumulation and retention. It has also been shown that, using nano-based systems, the adverse effects induced by standard contrast agents (i.e., contrast-induced nephropathy) are not observed [19]. For these reasons, metal nanoparticles are considered as a valid alternative to traditional contrast agents.

As for techniques based on luminescence, important results are achieved with semiconductor and upconverting nanocrystals. Indeed, these nanomaterials can show luminescence emission properties that can be tuned from the visible to infrared region, by varying the structural composition and the size of the particles; the luminescence properties are significantly superior to those of classic organic fluorophores.

**Scheme 1:**
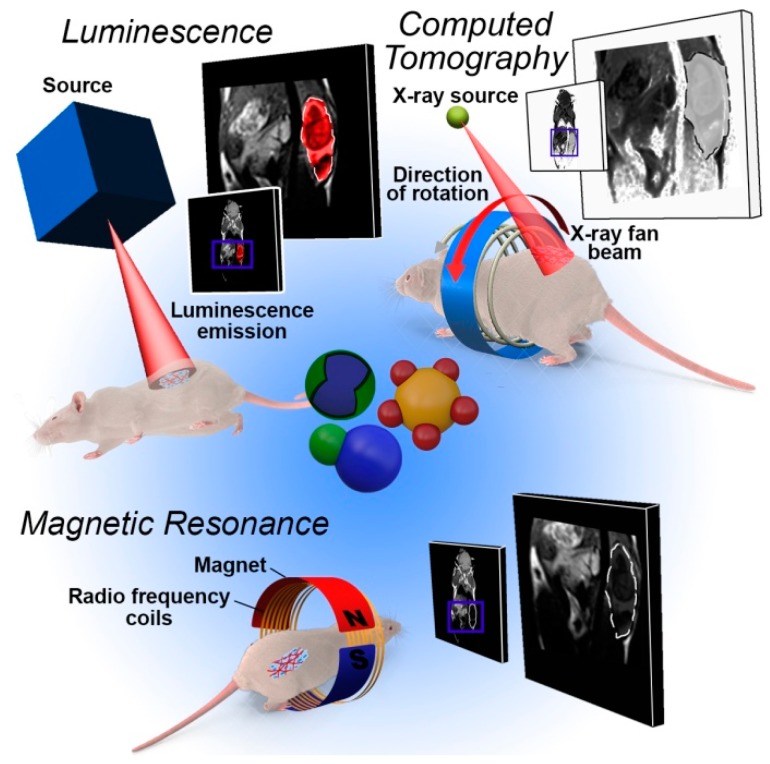
Overview of the imaging techniques most used in heterostructures-based diagnostics. Owing to the multiple domains structure, the patient model can be examined by different imaging methods after administration and delivery of a single nanostructure.

As mentioned above, more recently, bimodal imaging inorganic nanostructures have been developed, by combining luminescent and magnetic materials in core@shell geometries; these nanostructures can respond simultaneously to optical and MRI detection. Scheme 1 shows how a single nanostructure can be used in different imaging techniques, due to presence of multiple domains. 

In the next sections, a brief introduction of each technique is reported, explaining the physical principles behind it, followed by a detailed analysis of the NHS developed for the application in each field.

### 2.1. Bioimaging Techniques

#### 2.1.1. Magnetic Resonance Imaging

Magnetic resonance imaging (MRI) is a non-invasive technique based on the principles of nuclear magnetic resonance. More specifically, the signal recorder is related to the magnetic relaxation of the hydrogen nuclear spin of the water molecules in the body tissue. When an external magnetic field (*B*_0_) is applied, the nuclear spin aligns to the field, leading to a net magnetic moment (*m*). If a radiofrequency pulse (RF) perpendicular to *B*_0_ is applied, the spins start a rotation motion (precession) in a plane perpendicular to *B*_0_. The frequency of RF *ω*_0_ is related to the applied field *B*_0_ according to the formula,

$$\omega_{0}=\gamma \cdot B_{0}$$

where *γ* is the gyromagnetic ratio (42.6 MHz for ^1^H), which in turn is related to the frequency of Larmor precession.

Removing the RF, the spins recover gradually the initial state, parallel to *B*_0_, continuing their rotational motion. The recovery of this equilibrium is governed by two mechanisms, called relaxations: longitudinal relaxation process (*T*_1_) that follows the recovery of the magnetic moment along the *B*_0_ direction, and the transverse relaxation process (*T*_2_) that follows the loss of signal in the perpendicular plane to *B*_0_. The time lapses involved in these mechanisms correspond to the relaxation times (*T_n_*); these can also be expressed in term of the relaxivities (r_n_ = 1/*T_n_*). The latter is commonly expressed in mM^−1^·s^−1^ as a normalized unit.

The main critical issue of a standard MRI analysis is its relatively low resolution, due to the low contrast of the analyzed tissue. To overcome this limitation, specific structures showing enhanced contrast have been developed. Most MRI contrast agents work by shortening the relaxation time of protons located in the surrounding regions; this results in an “image” in which the morphological details are more defined [20].

The contrast agents in MRI can be classified as *T*_1_ and *T*_2_ agents; *T*_1_ contrast agents, generally based on Gd chelates, are paramagnetic ion metals with unpaired electrons in the outer orbital shells. When a magnetic field is applied, these ions generate strong magnetic dipoles that induce a faster *T*_1_ relaxation of close water protons, resulting in positive contrast enhancement of the imaged region. On the other hand, magnetic nanoparticles behave as *T*_2_ agents; indeed, their injection in the tissue under investigation leads to an additional magnetic field (when *B*_0_ is applied), which causes inhomogeneity in the field itself. Consequently, the transverse relaxation process is accelerated (with shorter *T*_2_ and higher r_2_ values); this causes an enhanced negative contrast [21].

In recent years, several formulations based on superparamagnetic nanoparticles have been tested as contrast agents for MRI and clinically approved, such as Cliavist/Resovist (Bayer Schering Pharma, Berlin, Germany), Combidex (AMAG Pharma, Waltham, Massachusetts, USA), Endorem and Sinerem, (Guerbet, Villepinte, France). Endorem™ for instance is one of the first commercially available compounds and it consists of an aqueous colloidal suspension of superparamagnetic iron oxide nanoparticles [22]. The particles, obtained by co-precipitation and coated/coordinated by a layer of dextran, display an inorganic core of about 10–20 nm in diameter and a final hydrodynamic diameter close to 120–200 nm. These formulations have been used in clinical diagnosis to detect liver lesions. Indeed, 5 min after their intravenous administration, they are retained by the liver; the macrophages localized in the healthy regions of the liver capture the iron oxide nanoparticles, whereas the damaged region is not stained because of the absence of macrophages. This leads to a decrease of the *T_2_* value only in the normal tissue; consequently, the lesions can be recognized. The main limitation of this product is that it is only effective for diagnosing liver disease due to the sequestration effect, caused by the low colloidal stability of the nanoparticles. To address this issue, in recent years, there have been several clinical trials using ultra-small superparamagnetic nanoparticles; these systems are considered to try to enhance the stability of the particles in biological fluids and increase their circulation time (clinical trials No. NCT00147238, NCT01270139, NCT01895829, and NCT02226523; www.clinicaltrials.gov).

In the synthesis of magnetic material-based NHS for MRI, several routes are considered; the prepared heterostructures have the following features/composition:

(i) Presence of two magnetic domains that cooperate to enhance the particle efficacy (increase in r_2_ value). This approach is generally typical of alloys for single-domain particles, but core@shell heterostructures based on two different magnetic materials have also been successfully prepared.
(ii) Presence of magnetic and metallic domains: their combination leads to higher MRI performance.
(iii) Presence of domains fulfilling different tasks: the magnetic ones serve for MRI, whereas the second material (metallic or semiconductor) accomplishes a distinct function. In the latter case, the system would be multifunctional; there is, however, the risk that the second domain may lead to a decrease of the MRI performance, owing to interferences with the magnetic field.

#### 2.1.2. Computed Tomography

Computed tomography (CT) is an X-ray based whole body imaging technique, widely used in medicine. A CT scanner is made of an X-ray source and a detector array. X-rays emitted from source penetrate the patient and are partly absorbed by the tissues. The radiation that passes through the body reaches the X-ray detector and is recorded. Generally, both the source and the detectors rotate around the patient in a synchronized motion, to build a three-dimensional reconstruction of the analyzed anatomical part through computer-assisted algorithms. The nature of elements in the analyzed portion of the body represents a key point to obtain images with high contrast. Soft tissues (as well as air) are made of elements with similar atomic number and k-edges (H, C, O, and N), leading to a low attenuation of X-ray. Harder tissues such as bones are rich in relatively high atomic number elements, such as calcium and phosphorous. Therefore, the resulting images show a high attenuation.

To obtain a better reconstruction, some CT contrast media are based on elements that have much higher Z values than physiological ones. At present, the approved contrast agents are iodinated compounds or barium-based formulations [23]. Recently, big efforts have been made to develop innovative contrast agents based on nanoparticles or ultrasmall structures; one of the reasons for this is that traditional contrast agents lead to an increasing number of renal insufficiency-based pathologies. The hypersensitivity of many patients to iodinated contrast agents is also another important factor [24]. In this frame, nanoparticles provide several advantages in terms of stability and functionality. NP-based agents are composed of different elements, including gold, silver, bismuth, bromine, tantalum, platinum, ytterbium, yttrium, gadolinium and tungsten. The main drawback of these elements is represented by their inner toxicity; to address this, several strategies have been adopted to reduce adverse effects. The inclusion of the NPs in core@shell systems is a possible option, i.e., liposome, emulsions, silica shells or polymeric nanoparticles [25]. Moreover, hybrid NHS have been developed; they are based on the combination of different domains, thus exhibiting different functionalities and being exploitable in CT and other diagnostic or therapeutic techniques.

#### 2.1.3. Luminescence Imaging

Luminescence imaging is a versatile and easily accessible technique for cellular and preclinical studies, with remarkable performance, due to significant advancements in instrumentation and probe. Luminescence is generally defined as the emission of light by molecules/materials, whose excitation sources fall within the electromagnetic spectrum. Compared to other imaging techniques, it is generally easier to use, it does not involve ionizing radiation and it is non-invasive; moreover, it has good sensitivity, as well as good spatial and temporal resolutions.

It is worth reminding that in vivo imaging is more challenging if compared to in vitro processes, due to the diffusion and absorption of the light by the tissues and to the autofluorescence emitted from intrinsic molecules. The near-infrared region of the spectrum offers deeper photon penetration and reduced scattering in comparison to the ultraviolet excitation source. In addition, some imaging setups require multiplexing analysis, crucial for the simultaneous recording of multiple events or for the real-time labeling of different compartments and biological targets [26].

To address these issues, innovative imaging probes have been developed in the last 15 years, as an alternative to common organic fluorophores. Indeed, two important classes of luminescent nanocrystals—semiconductor quantum dots and upconverting nanocrystals—have been considered due to their robustness, their displaying superior optical properties in terms of large Stokes shift, high quantum yields, photostability and their reduced tendency to photo-oxidation. Moreover, their high surface to volume ratio allows an easier surface functionalization and conjugation of selected biomolecules, for specific targeting. All these features lead to the development of these inorganic fluorophores as versatile in vitro and in vivo tools for labeling and imaging in life science.

Considering more in detail these two classes of nanomaterials, colloidal quantum dots (QD) are semiconductor nanocrystals, few nanometers in size; they are made with atoms from Groups II–VI, III–V, or IV–VI of the periodic table [4]. Their unique optical properties arise from their peculiar electronic structure and from their dimensions close to or smaller than the exciton Bohr radius. In these conditions, the electrons and hole carriers are confined in discrete energy bands and the absorbance of energy higher than the band gap leads to the creation of an electron–hole pair, an exciton. The radiative recombination of the exciton generates the QD photoluminescence; this depends on their size, as a consequence of the quantum confinement. The confinement of the electron–hole pair in smaller QD corresponds to higher energy band-gap, which results in emission at higher frequency (in the blue region); for larger QDs, on the other hand, emission is at lower energies (in the red or NIR region). QDs exhibit interesting properties, such as high quantum yield, high molar extinction coefficients, narrow luminescence spectra, large Stokes shifts, and high resistance to photobleaching [27]. Moreover, since QDs show broad absorption spectra, this allows the excitation of multiple QD at a single wavelength, which enables multiplexing analysis. Due to their significant performance, QDs have been employed in biological studies, from single molecule tracking to cell labeling, from gene delivery to in vivo imaging [28,29].

Considering lanthanide-doped upconverting nanocrystals, some of their most interesting features include the efficient upconversion photoluminescence and the resistance to photoblinking and photobleaching. Moreover, the upconversion mechanism is based on the use of NIR excitation; this overcomes the issues related to radiation absorbance and autofluorescence of tissues. Additionally, these systems enable multimodal imaging, due to the possibility of doping with appropriate ions, which can be exploited in MRI, CT or PET (i.e., Gd^3+^, Lu^3+^ and ^153^Sm^3+^, respectively) [30].

In detail, the process of photon upconversion (UC) is characterized by the conversion of long-wavelength radiation, such as infrared or near-infrared (NIR), to short-wavelength emissions, such as visible or UV light. Thus, UC is a nonlinear optical process that consists in the excitation of lower electronic levels and in the following emission at higher electronic levels; this can be defined as an anti-Stokes mechanism. The processes governing the UC are quite complex and a detailed description is outside the scope of this work; in brief, for UC to take place, two or more photons have to be absorbed, while three different mechanisms (excited state absorption, energy transfer and photon avalanche) may be involved [31,32]. The UC process requires a host lattice—which determines the spatial distribution of the dopants, to enhance the fluorescence emission and to reduce energy losses—the sensitizer ions (i.e., energy donators), the activator species (i.e., radiation emitters) and an appropriate excitation source. Thus far, several types of host materials have been developed such as NaYF_4_, NaYbF_4_, and LaF_3_, doped with different ions, such as Er^3+^, Tm^3+^ or Yb^3+^ [33].

#### 2.1.4. Photoacoustic and Photothermal Imaging

In this section a brief description of photoacoustic and photothermal imaging techniques is given. Although these methodologies are not the main scope of this review, some of the NHS described hereafter also show this imaging functionality.

The photoacoustic (PA) imaging technique is based on the photoacoustic effect, i.e., the generation of sound wave by a sample after irradiation with a suitable light source. More specifically, a laser with wavelength > 650 nm is generally used as a source; some molecules in tissues can generate the sound wave signal, which can be registered and successively converted into a spatially resolved image [34]. To enhance such effect, and hence the sensitivity of the technique, appropriate contrast agents are normally used; NHS can be very suitable for this [35].

Photothermal (PT) imaging, on the other hand, is based on the increase in temperature caused by the absorption of light. The higher temperatures lead to different optical properties (i.e., refractive index), which can be detected and converted into an image [36,37]. As stated above for PA, NHS can be employed as effective contrast agents.

### 2.2. Nanoheterostructures for Diagnostic Imaging

In this section, the nanoheterostructures for diagnostic imaging are presented: depending on the composition of the inorganic domains, the NHS are divided in two categories: magnetic/metallic and luminescent NHS. The latter are nanomaterials composed of at least two domains, one of them being luminescent. Several examples of NHS for diagnostic imaging in vivo are described, focusing on those that have been applied for in vivo imaging, as reported in Table 1.

#### 2.2.1. Magnetic/Metallic Nanoheterostructures

For the NHS, the most used architecture is the core@shell: in a typical approach, a magnetic core, mainly made of iron oxide, is coated by an outer shell of metallic material. With this design, the magnetic properties of the core are slightly affected by the shell (with the exception of the application that require a Brownian contribution, e.g., magnetic hyperthermia). At the same time, the metallic shell can perform its function, without any quenching or partial absorption of the excitation radiation due to the magnetic domain. Kumagai et al. reported the synthesis of a high-density poly(ethylene glycol)-coated iron-oxide@gold core@shell nanoparticles. The gold shell was introduced to provide an efficient surface coating to the particles and therefore an increased stability in the bloodstream. A standard sulfur chemistry was used to anchor the PEG to the shell, obtaining an overall diameter of 25 nm. The particles showed a r_2_ value of 149 mM^−1^·s^−1^, comparable to that of commercial standard Feridex. Biodistribution experiments were performed in mice bearing a subcutaneous colon cancer model. After intravenous injection, the accumulation of NPs (via gold detection) was monitored in tumor and organs; results indicate a high specificity for solid tumors. MRI images confirmed the biodistribution results; the use of these structures led to a selective negative enhancement of tumor tissue at 4 h post-injection time [38].

Following a different synthetic approach, Zhou et al. described the synthesis of a multimodal nanostructure based on magnetic nanoparticles prepared by co-precipitation method. The nanoparticles, initially stabilized by a layer of sodium citrate molecules, were successively covered by a gold shell, obtained adding chloroauric acid. The Fe_3_O_4_@Au NPs were further functionalized with mercapto-undecanoic acid, PEG and FITC-labeled integrin α*_v_*β_3_ mAb; this was done to achieve a specific recognition of receptor overexpressing U87-MG cancer cells. The diagnostic efficacy of these nanostructures was evaluated by both microwave-induced thermoacoustic imaging and photoacoustic imaging. The gold shell and the subsequent functionalization did not affect the MRI performance, obtaining a r_2_ value of 26 mM^−1^·s^−1^, slightly lower than citrate-coated magnetic particles (36 mM^−1^·s^−1^) [61].

**Figure 1:**
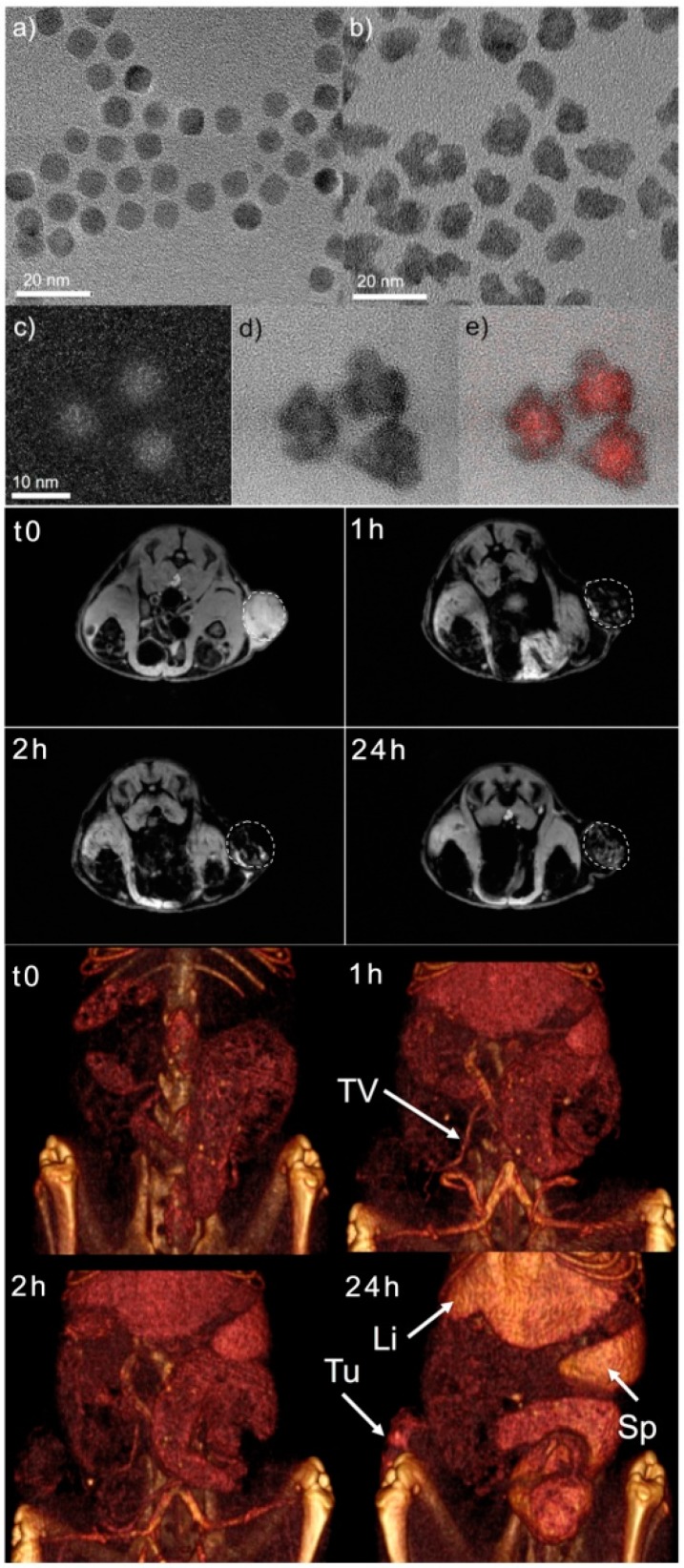
Fe_3_O_4_@TaO_x_ core@shell NPs for dual imaging. (**a**,**b**) TEM images of magnetite NPs before and after the growth of tantalum oxide shell. (**c**–**e**) The panel (**e**) shows the overlay of the iron elemental analysis (**c**) and the corresponding bright field image, highlighting the core@shell architecture of the nanostructure. The central panel and the lower one represent the imaging of a rat after administration of the heterostructure, following the intensity of the signal during a time lapse of 24 h. In MRI, the tumor (dashed lines) became darker after a short time from the injection, whereas for CT-imaging the accumulation in different organ was detected during the observation. TV, Li, Tu, and Sp indicate the tumor-associated vessel, liver, tumor, and spleen, respectively. Adapted with permission from *J. Am. Chem. Soc.* **2012**, *134*, 10309−10312 [39]. Copyright 2012 American Chemical Society.

Tantalum-based coatings are also widely employed for the outer shell of magnetic nanoparticles. In 2012, Lee et al. made a multimodal imaging system combining an iron oxide core (Fe_3_O_4_) with a tantalum oxide shell. In this study, the shell was obtained by a sol–gel reaction, using oleic acid-capped magnetic nanoparticles in a microemulsion of Igepal CO-520. The addition of tantalum(V) ethoxide at room temperature resulted in the quick formation of TaO_x_ shell. The NPs were tested for MRI (r_2_ of 81 mM^−1^ s^−1^) and CT imaging, giving a significant signal for both techniques. Moreover, the nanoparticles were injected intravenously in rats bearing xenograft tumors; this allowed the detection of the vascular system of the tumor. More specifically, the tumor-associated vessels were observed using CT, while MRI revealed the high and low vascular regions of the tumor (Figure 1) [39]. More recently, the same authors measured quantitatively the contrast enhancement produced by these particles, with images acquired by MRI and micro-CT scans after intravenous injection of Fe_3_O_4_@TaO_x_ nanoparticles into the tail. To assess the extent of the contrast enhancement, images were acquired pre- and post-injection and the differences were considered. As expected, the nanoparticle uptake at the tumor site and vasculature increased with time, reaching a maximum uptake ration of 0.16 after 24 h. In the MR images, the tumor region was distinguishable soon after 5 min post injection, while in CT image only after 24 h [40].

In the framework of core@shell particles, Li et al. reported the synthesis of novel Fe_3_O_4_/Gd_2_O_3_ nanocubes as MRI *T*_1_/*T*_2_ contrast agents. The co-localization of the two materials in a single heterostructure offers the possibility of a complementary diagnostic analysis to overcome the limitations of single *T*_1_ or *T*_2_ imaging. These nanoparticles were obtained by a one-pot synthesis, starting from metal oleate precursors. At 1.5 T the relaxivity values were 45.24 mM^−1^·s^−1^ and 186.51 mM^−1^·s^−1^ for r_1_ and r_2_, respectively. The consequently low r_2_/r_1_ ratio allowed using this contrast agent in MRI for positive and negative contrast, as evaluated in mice liver after intravenous injection [41]. Moreover, Tang et al. reported the synthesis of a core@shell nanoparticle based on a core of iron carbide Fe_5_C_2_ (19 nm) and a thin shell of Fe_3_O_4_ (less than 2 nm), with an overall size of 23 nm. These nanoparticles displayed exceptional r_2_ relaxivity, close to bulk values, of 464 mM^−1^·s^−1^, which is among the highest of all MR probes reported. After functionalization with the c(RGDyK) peptide (a RGD derivative with high affinity towards α_v_β_3_ integrin and overexpressed on tumor vasculature and various types of tumor cells), the nanoparticles were injected intravenously (i.v.) in mice for the identification of inducted subcutaneous tumor. Compared to RGD-Fe_3_O_4_, RGD-Fe_5_C_2_ induced 42.6% and 60.7% greater signal at 4 and 24 h time points, respectively [42].

The design of a nanostructure should be tailored according to its functional use; thus, a peculiar geometry derived by the combination of different materials could contribute to introduce additional properties. In this contest, asymmetric NHS, in which two or multiple domains are geometrically associated, have been developed. Yang et al. reported the synthesis and functionalization of highly monodispersed heterostructured NPs, having both a magnetic and a plasmonic domain. The NP were obtained via solvothermal method, by injection of iron pentacarbonyl for nucleation of magnetic particle on pre-synthesized gold seeds. The authors aimed at obtaining a multimodal nanoprobe employable for PET, optical and MR imaging. For this purpose, the magnetic domain served as a *T*_2_ reporter for MRI, whereas gold component served as both optical and PET reporters. The surfaces of the two domains allowed selective functionalization with targeting molecules (anti-EGFR protein) and PET reporter (radiometal ^64^Cu chelators). MRI analysis revealed that Au-IONPs heterostructure had a valid performance as *T*_2_ agent, with a r_2_ relaxivity of 143.2 mM^−1^·s^−1^, which was close to that of the commercial MRI contrast agent Feridex (158.3 mM^−1^·s^−1^). The dual imaging capability of the nanostructure was also tested in vivo in mice bearing A431 tumors. For MRI, at 4 h after tail vein injection, the tumor signal was darker than that of the pre-injection group. At 48 h from injection, a signal 44% weaker in comparison to controls group (non-treated or injected with anti-EGFR antibody) was detected. The results were confirmed also with PET imaging, observing a gradual increase of the intratumoral signal exclusively in treated group [43].

Considering a similar approach, Zhu et al. reported the synthesis of bifunctional Fe_3_O_4_-Ag^125^I heterodimers as dual imaging agents, combining MRI to single-photon emission computed tomography (SPECT). Firstly, iron oxide nanocubes were synthesized, followed by the growth of the silver domain. After transferring in water, Fe_3_O_4_-Ag NHS were mixed at room temperature with the radionuclide sodium iodine-125 (^125^I): the silver domain served as template for the incorporation of ^125^I [44]. In parallel to this work, the same research group reported the synthesis of iron oxide-gold heterostructure for in vivo imaging. The NPs were typically obtained by inducing the nucleation of a magnetic domain on pre-formed gold seeds, through the addition of iron pentacarbonyl as metallic precursor. The hydrophobic dimers were transferred in water by surface modification with tetramethylammonium hydroxide. The NPs displayed an overall inorganic diameter of 25 nm (11 nm for gold seed and 14 nm for IONP), with a r_2_ value of 136 mM^−1^·s^−1^. For in vivo analysis, the heterodimers were injected in the ear vein of rabbits, and major organs, such as heart and liver, were monitored, respectively, by CT and MRI. The CT signal reached the maximum at 25 s post-injection and decreased rapidly, due to fast blood dilution. The accumulation in liver took longer, achieving a decreased signal from 5 to 10 min in MRI *T*_2_-weighted sequences [45].

Efremova et al. reported the synthesis of a magnetite-gold nanohydrid. The nanostructure was composed of 25 nm octahedral-shaped Fe_3_O_4_ magnetite nanocrystals epitaxially grown on the surface of Au seeds. The hybrid was first stabilized with oleic acid and then transferred in water by lipidic layer. The unusual shape led to superior in vitro and in vivo *T*_2_ relaxivity compared to commercial agents (r_2_ in H_2_O was 498 mM^−1^·s^−1^, in 4T1 cells was 277 mM^−1^·s^−1^). Moreover, the gold seed was exploited as a platform for the selective conjugation of dyes or chemoterapeutic drugs [46].

**Figure 2:**
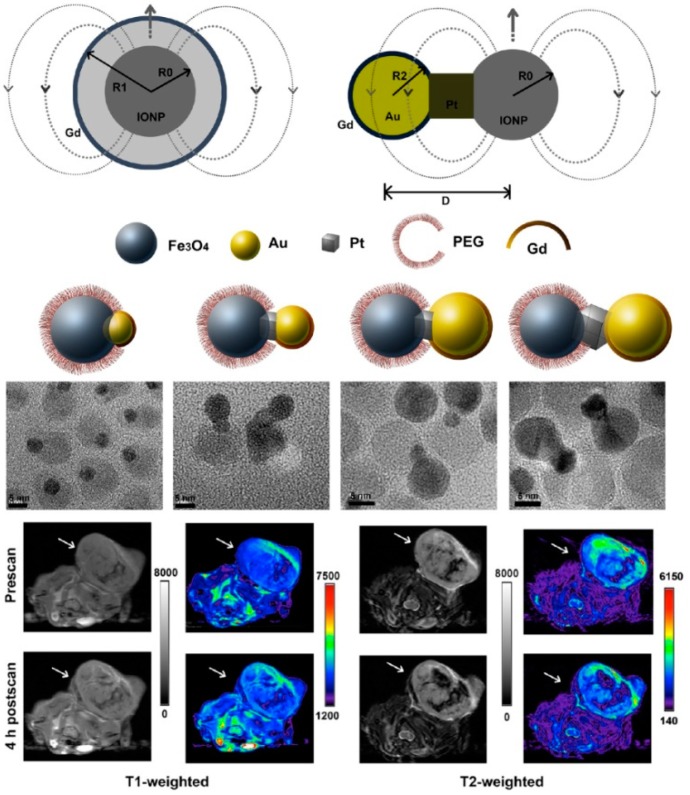
Design of a trimeric dumbbell for *T*_1_/*T*_2_ MRI analysis. The coating of iron oxide NPs with Gd-chelates hampers the exploitation as *T*_1_ imaging probe, due to the close proximity of the magnetite core. The presence of the Pt spacer between iron oxide and gold NPs allows increasing the distance between the two MR contrast agents (Gd-chelate anchored on gold domain) and to achieve an effective dual MR imaging. In the lower panel, MRI was performed before and 4 h after the injection of heterostructure. For each time point, gray scale and pseudo-colored images are reported. Adapted with permission from *ACS NANO* **2014**, *8*, 9884–9896 [47]. Copyright 2014 American Chemical Society.

Recently, Cheng et al. introduced a third metallic domain to enhance the properties of magnetic-gold heterodimers. The authors obtained a trimer nanostructure, combining iron oxide, platinum and gold domains through a two-step synthesis. First, iron oxide was nucleated onto Pt cubic seeds; then, gold NP grew on Pt facets of IONP-Pt dimers. By tuning the Pt seed size and the amount of gold precursor, the authors prepared several NHS with different distances between the domains and the exposed active surface. In this trimeric structure, the Pt domain acted as an inorganic spacer between the two active agents, i.e., the magnetic one, as *T*_2_ contrast agent in MRI, and the gold one, as support for PET reporter ^64^Cu and for Gd-chelates (*T*_1_ agent). By increasing the distance between *T*_1_ and *T*_2_ species, the magnetic coupling was reduced and both *T*_1_/*T*_2_ contrast effects were enhanced. The most performing nanostructures were injected in HT-29 tumor bearing mice and analyzed by MRI, PET and CT. The three imaging techniques confirmed the specific accumulation of the trimers in the tumor stroma and the multivalent imaging performance by means of PET/CT and *T*_1_/*T*_2_ MRI (Figure 2) [47].

The synthetic approaches based on the solvothermal method are particularly suited to obtain multi-domain NHS with nature-inspired geometries. An example of these geometries is the flower-like shape of some heterostructures. Kim et al. reported the preparation of multicore NHS based on gold and iron oxide nanoparticles. The metallic precursors reacted with sodium oleate or oleylamine to form, respectively, reactive Fe-oleate and Au-oleylamine complexes. The species were then mixed, obtaining a non-regular structure with an average diameter of 20–25 nm, which consisted of the gold domain at the center and multiple magnetic nanoparticles at the outer part of the structure, in a flower-like structure. Using a nano-emulsion method, the nanoparticles were coated with an amphiphilic polymer, containing repeated PEG moieties, to impart water-dispersity and antibiofouling properties. Interestingly, the MRI performance of the heterostructure was enhanced, likely due to a coupling effect of the multiple domains, leading to a r_2_ value of 245 mM^−1^·s^−1^. In vivo imaging studies were performed by injecting the NPs trough the tail vein of hepatoma-bearing mice. The biodistribution and the uptake of the hybrid nanoparticles by the liver macrophages were monitored along a 24 h time-lapse post injection; after 1 h, an effective CT and MR contrast was detected [48].

In the same year, Xie et al. reported the synthesis of a flower like gold-magnetic heterostructure. Even in this case, the gold nanoparticles served as internal core and the magnetic nanoparticles were subsequently grown on the gold surface, as petals. A NIR emitting fluorophore was then anchored to the magnetic surface; the fluorescence quenching (due to gold proximity) or lighting (after release of the fluorophore from the magnetic domain) were evaluated under the effect of enzymatic activity. More specifically, the magnetic nanoparticles were functionalized with a formulation made of TDOPA (dopamine derivative), a sequence sensible to metalloprotease (MMP), and the NIRF dye Cy5.5. When linked to the nanoparticles, the fluorophore is quenched due to gold proximity, but in MMP rich tissues, such as tumors, the TDOPA formulation will be cleaved, thus releasing the dye; this, in turn, recovers the fluorescence emission. This model of the activation of the fluorescence mediated by protease activity was evaluated in vivo in mice bearing a SCC-7 (head and neck squamous cell carcinoma) tumor: the NIRF intensity was monitored in a 4 h time lapse. A strong fluorescence was detected in the tumor matrix, whereas no phenomena were detected in control group with MMP-inhibitors. The presence of NPs and Cy5.5 in tumor was also confirmed by ex vivo histological analysis and by Prussian Blue staining and fluorescent immunostaining, respectively [49].

#### 2.2.2. Luminescent Nanoheterostructures

As mentioned in the previous sections, several imaging techniques are routinely used in research and clinic, including CT, MRI, PET, SPECT, ultrasound, and optical imaging, each displaying advantages and limitations, depending on the imaging principle and the biological target under analysis. Among them, MRI is considered to be superior to acquire anatomical and physiological images with high spatial resolution, while optical imaging is more suitable for translation into the clinics, thanks to its high sensitivity and the lower cost of the imaging facilities.

In this context, bifunctional magnetic and luminescent probes allow the combination of imaging modalities and the improvement of the diagnostic efficacy. Nanostructured multimodal imaging probes for simultaneous MRI and optical imaging are the most studied in lab-scale research, since they provide high spatial resolution (through MRI) and rapid optical screening of the target site.

In literature, several methods for the preparation of hybrid magnetic-fluorescent nanoparticles have been described. Among them, those based on direct conjugation of the two domains (i.e., encapsulation within a host material, and ion doping as in the case of the rare earth-doped upconverting nanoparticles) are the most frequently presented; however, they are not considered within this review as it focuses on inorganic heterostructures.

Magnetic-fluorescent NHS are generally made of core@shell nanoparticles or heterodimers, in which the magnetic domain, made of iron oxide nanoparticles (maghemite or magnetite), is associated to the fluorescent ones, consisting of either of the “classic” semiconductor quantum dots or, in more recent works, of upconverting nanoparticles.

In 2008, Fe_3_O_4_-CdSe heterodimers were developed by using 4 nm Fe_3_O_4_ nanoparticles as seeds to grow CdSe QDs [62]. The derived heterodimers exhibit both fluorescent and superparamagnetic nature. The presence of the metal oxide domain significantly affects the photoluminescence efficiency, as expected by the close proximity of the two domains. The biological testing of the NHS is limited to confocal imaging of human cells (HEK293T, originally derived from human embryonic kidney cells); however, this study deserves to be reported as it is one of the first examples of biological application of inorganic heterostructures. In detail, the tests were performed to evaluate the magnetic responsiveness of the nanoparticles once internalized, and their intracellular localization. Prior to cellular studies, the nanoparticle surface was modified with glutathione, which is known to accelerate NP entry through the GSH receptor that is expressed on the membrane of replicating cells. When the cells were kept under a magnetic field for a continuous period of 8 h, the internalized nanoparticles appeared localized in large clusters in the cellular region closest to the magnet, rather than being distributed randomly.

In the same period, the group of Louie [63] reported one of the first examples of magnetic-fluorescent core@shell quantum dots with dual imaging properties. CdSe quantum dots were coated with a paramagnetic Zn_1−x_Mn_x_S shell (Zn-doped MnS shell). This system cannot be considered a “true” heterostructure, but rather an example of ion-doped heterostructure that combines the benefits of the dual functionality with those arising by the growth of a protective shell over QD core. Indeed, the presence of the shell improved QDs photostability from degradation and bleaching, while at the same time imparting the magnetic functionality. The core@shell nanoparticles were made water-dispersible by wrapping them with octylamine-modified poly(acrylic) acid. The water solubilization affected the QY efficiency, which decreased from 45% to 15% (average values), as already reported in previous studies [64,65]. The dual imaging properties of the core@shell QDs were demonstrated in vitro. *T*_1_-weighted MR and fluorescent images of the QDs solution confirmed the bimodal nature of the heterostructure, while confocal imaging studies in macrophages showed the typical endocytic localization of QDs, as evidenced by the point-like distribution of the fluorescent signal. The same cells incubated with QDs were also imaged by MRI. *T*_1_-weighted images showed a modest contrast enhancement as compared to control cells.

Since then, other examples of bimodal QDs based on doped shell geometries have been reported [50,51,66]. In two more studies from the group of Chen, QDs act as energy acceptors in the presence of an internal light source through the Cerenkov resonance energy transfer (CRET) mechanism; such mechanism links the radionuclide imaging to optical imaging. More specifically, upon radioactivity decay, the light emitted from β- and α-emitting radionuclides can be used to excite QDs photoluminescence. In both studies, radioactive ^64^Cu was incorporated during the growth of the QD shell through cation exchange reaction, leading to heterostructures for dual in vivo imaging via PET and photoluminescence emission. In the first work [50], ^64^Cu-doped CdSe@ZnS QDs were developed; they were tested for dual imaging modality with in vitro phantoms. This was done with intravenously injection (25 μg QDs that correspond to 250 μCi ^64^Cu) in a U87MG glioblastoma xenograft model. Whole-body PET and luminescence images were recorded at 1, 17, 24, and 42 h post-injection. Liver, spleen and the tumor region were clearly visible reaching about 10% ID/g uptake and retention of the signal at tumor after 42 h. In the second study [51], [^64^Cu] doped CuIn@ZnS QDs (with photoluminescence peaking at around 670 nm) were synthesized and functionalized with methoxy-PEG-thiol and GSH prior to cellular and animal testing. The same type of xenograft tumor model was employed, as well as intravenously injection with the radioactive QDs. Successful PET and Cerenkov luminescence were reported, with a maximum uptake of the nanoparticle at the tumor equal to 10.8% ID/g at 18 h post-injection; this was followed by a slow decrease at 48 h, likely due to nanoparticle washout and clearance.

With the increasing interest in upconverting (UC) luminescent nanoparticles as new generation of inorganic fluorophores, several studies attempted to develop magnetic-fluorescent nanostructures. In these systems, the fluorescent domain is made of UC nanoparticles, whose emission window in the visible-NIR region can be adjusted by varying the material composition, and thus adapted to the application devised. The magnetic domain, on the other hand, generally consists of superparamagnetic iron oxide nanocrystals.

A NHS with these characteristics was developed in two distinct works by the group of Fuyou Li [52,53] and in the study of Yi Jin [54]. In the first study, NIR-to-visible/NIR upconverting NaYF_4_:Yb^3+^,Tm^3+^ cores were synthesized via thermal decomposition method and then iron oxide shells were grown on them, leading to uniform 30 nm sized core@shell fluorescent-magnetic nanostructures. Under continuous wave excitation at 980 nm, the nanoparticles displayed upconverting emission peaks at 452, 475, 650, 700 and 800 nm, being the emission curves in the red-NIR region less affected by the presence of the superparamagnetic shell, as expected. The nanoparticles were transferred from cyclohexane into water by exchange of surfactant molecules with dopamine. Then, upconverting luminescence imaging in vitro was carried out upon incubation of human carcinoma cells (KB) with NaYF_4_:Yb^3+^,Tm^3+^@Fe_x_O_y_ nanoparticles; emission signals were collected at 450–500 nm and at 980 nm under excitation. Due to excitation and emission windows in the NIR region, the NHS were applied for in vivo imaging of the lymphatic system of murine models. To this aim, NaYF_4_:Yb^3+^,Tm^3+^@Fe_x_O_y_ nanocrystals were injected into the right forepaw of a mouse, and the spatial distribution of the upconverting luminescence signal in the lymphatic vessel was monitored over time. After one week, the signal was still detectable in the axillary lymph node and in the forepaw. Similarly, the MR susceptibility of the heterostructures was investigated in vivo on a 3T MRI scanner. Preliminary *T_2_*-weighted images of nanocrystals solution at increasing concentrations of particles were collected; results showed that, despite the small thickness of the iron oxide shell (around 5 nm), the negative contrast enhancement obtained was almost two times higher than that of conventional Feridex particles. Upon injection of the NHS in the lymph node of the mouse forepaw, MR imaging was recorded, revealing about 50% enhancement of the contrast signal after 30 min.

To reduce the optical quenching due to the magnetic domain, the same group proposed a new system in which the core was made of iron oxide and a rare earth upconverting shell, made of NaLuF_4_ doped with Yb and Er or Tm, was grown on top [53]. This multifunctional mesostructure displayed a final size of 300 nm, with the magnetic core of around 180 nm; it was prepared by a step-wise method that included the synthesis of a temporary silica shell between the magnetic and the upconverting domains. Indeed, after the HF treatment needed to convert Fe_3_O_4_@SiO_2_@Lu_2_O_3_:Yb,Er/Tm into Fe_3_O_4_@NaLuF_4_:Yb,Er/Tm, the silica shell was almost completely removed, thus generating a hollow structure. Though this complex system cannot be considered an ideal heterostructure, it is worth mentioning it as an example of reverse configuration, which displays the magnetic component inside and the fluorescent ones outside. Moreover, thanks to the high X-ray absorption coefficient of Lu, Fe_3_O_4_@NaLuF_4_:Yb,Er/Tm particles could also be applied as CT imaging probes. The trimodal imaging properties of the heterostructure were demonstrated both in vitro and in vivo upon intra-tumor injection of the nanoparticles in nude mice bearing xenograft tumors. Besides the quantitative data, the possibility to detect injured tissue(s), organ lesion(s) or tumor site(s), as in this specific case, with three imaging techniques allows a deep spatial and anatomical resolution and a visual accuracy with important diagnostic implications. Clearly, the large size of these particles represents a crucial limitation that prevents their potential application on humans; on the other hand, the performances provided by these particles show the intrinsic potential of the multifunctional systems.

Recently, examples of theranostic nanostructures based on magnetic/optical upconverting core@shell NHS are also published. In these works, besides the dual imaging performance, the nanoparticles are also investigated as chemosonodynamic [54] and photodynamic therapeutic [56] agents, respectively. It is worth mentioning that, in both studies, the therapeutic potential is not directly fulfilled by the heterostructure (hence, they are not included in the “theranostics” section of this review), but rather by a hosted drug or by the combination of the inorganic domain with the loaded drug, such as in the case of upconverting-mediated photodynamic therapy (PDT). Indeed, rare earth NIR luminescent nanoparticles conjugated with photosensitizers have emerged as new systems for PDT, thanks to deep tissue penetration of the excitation source, to low tissue autofluorescence in the NIR region, and to the possibility to match the emission of UC nanoparticles with the excitation wavelength of photosensitizers [55,67].

Zeng et al. prepared core@shell Fe_3_O_4_@NaYF_4_:Yb/Er nanoparticles and then electrostatically coupled them to AlPcS4 photosensitizer [56]. The as-prepared particles were tetragonal, with an average TEM size of 50 nm, and were coated with PEG for water solubilization prior to be conjugated to the photosensitizer. Cell viability studies were then performed to assess the cytotoxic effects of the nanocomposites under 980 nm laser irradiation. The UC emission in the 655–675 nm window excites the AlPcS4 photosensitizer that, in turn, generates cytotoxic singlet oxygen for PDT of cancer cells. Several conditions, such as irradiation time and nanoparticle concentrations, were tested to find the highest percentage of cell mortality.

More recently, the same group also added folic acid to this nanostructure to enhance the targeting capacity; these structures were applied for tests of in vivo tumor bearing mice [68]. After the introduction of MCF7 cells in the right flank of nude mice and the growth of the tumor mass, the nanocomposite was intratumorally injected. Besides the MR contrast enhancement of the tumor region, the UC-mediated PDT was assessed by measuring the fluorescence distribution of the photosensitizer and the variation of the tumor volume. After 15 days of treatment, the tumor volume significantly decreased as compared to animals not treated with PDT. Moreover, the biodistribution of the nanoparticles and their biosafety was assessed by injecting them in the tail vein of the mice and analyzing the major organs. The histological analysis did not show evident tissue lesions. The organ structure was similar to control mice and no sign of inflammation was detected. The nanoparticles biodistribution was followed by the luminescence of the upconverting shell: the nanocomposites mainly accumulated in the liver and at the tumor site, due to the sequestration by Kupffer cells in the liver and the enhanced permeation and retention (EPR) effect of nanomaterials in the tumor, respectively. Interestingly, folic acid functionalized nanocomposites accumulated at higher extent in the tumor, likely for their targeting capacity and their overexpression of folic acid receptors on the membrane of MCF7 cells. Finally, the histological analysis performed after NIR irradiation and PDT revealed the absence of any pathological effects on major organs (heart, liver, spleen, lung, and kidney), thus confirming the safety of the nanocomposites and of the treatment adopted.

**Figure 3:**
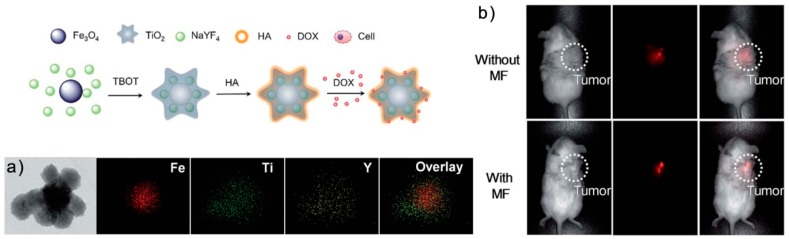
Sketch illustrating the synthesis of HA coated Fe_3_O_4_–NaYF_4_@TiO_2_ nanocomposites. (**a**) TEM and EDX maps of the distribution of Fe, Ti, and Y elements in the nanoparticles. (**b**) In vivo UCL images of the nanocomposites injected in tumor-bearing mice in bright field (left), dark field (middle), and overlay (right) under a magnetic field (bottom) and without a magnetic field (top). Adapted with permission from *J. Mater. Chem. B* **2014**, *2*, 5775–5784 [54]. Copyright 2014 Royal Society of Chemistry.

With an approach similar to PDT, sonodynamic therapy (SDT) utilizes sonosensitizers to generate ROS under ultrasound activation. The combination of this method with chemotherapy (multifunctional cancer treatment) was achieved through the development of dual core@shell Fe_3_O_4_–NaYF_4_@TiO_2_ nanostructures loaded with doxorubicin [54]. In this complex system, the cores made of either Fe_3_O_4_ or NaYF_4_ nanoparticles were separately synthesized. Then, the two kinds of nanoparticles were assembled; this could be done since they have opposite charge, i.e., negative and positive for the magnetic and upconverting, respectively. This structure was then used as template for the growth of the titanium dioxide shell, which displays sonodynamic properties [69]. Finally, the chemotherapeutic doxorubicin was adsorbed on the nanocomposite. Thus, the dual core@shell nanocomposite showed dual imaging properties by means of magnetic and upconverting nanoparticles, and dual therapeutic potential through chemotherapy and sonodynamic therapy. The effects of sonodynamic therapy combined to the release of doxorubicin were assessed in vitro, under the application of ultrasounds (1 W·cm^−2^). Cell viability assays, FACS analysis and monitoring of ROS generation showed the high cytotoxicity of the combined approach. To enhance the tumor targeting capacity, the surface of the nanocomposites was modified with hyaluronic acid (Figure 3). According to the authors, the functionalization would allow the nuclear targeting of the particles; considering the average hydrodynamic size of the nanostructure (around 400 nm), however, it is unlikely that such objects could overcome the nuclear membrane. On the other hand, it is more likely that the adsorbed doxorubicin, once the nanostructure has been endocytosed, escaped the endosome confinement and entered the nucleus. In this regard, it is worth remembering that, apart from the surface modification, other parameters such as the size, shape and charge of the particles play a crucial role in determining the interactions with the cells, their intracellular fate [70,71,72], and once in vivo, their circulation time and biodistribution [73,74,75].

Furthermore, these dual core@shell structures were injected intravenously for cancer targeting in tumor bearing mice. Under 980 nm laser excitation, the tumor region was imaged, showing enhanced accumulation of the upconverting luminescent signal (at 640–680 nm range) when a magnet was applied close to the tumor for 24 h. The biodistribution of the drug-loaded NHS was also analyzed and compared with that of free doxorubicin. The free drug reached the tumor faster and then was rapidly washed out from the renal system within the first 12 h; the drug associated to the multifunctional system, on the other hand, reached the tumor after the first 12 h and accumulated massively at the liver, showing negligible renal clearance. Finally, the synergistic therapeutic effect was determined: tumor-bearing mice injected with the doxorubicin-loaded particles and simultaneously exposed to ultrasound treatment showed the most consistent reduction of tumor mass and volume. These findings confirmed the current trends of the anticancer treatments that recommend the use of simultaneous therapeutic strategies to weaken tumor defenses and improve the chemotherapy activity.

In all magnetic/upconverting NHS presented thus far, the magnetic domain consisted only of superparamagnetic nanocrystals; there are, however, other studies based on different magnetic domains.

Recently, Shao et al., for instance, developed an UC fluorescent/paramagnetic core@shell heterostructure by growing a Gd-doped TbPO_4_ shell over a Ce-doped TbPO_4_ core via hydrothermal synthesis [76]. This system recalls the Mn-doped shell coated CdSe QDs that have been described previously [63]. As stated above, this cannot be considered a genuine heterostructure since it is not made of two distinct domains. Instead, it consists of a core@shell system in which the addition of a new element, Gd in this case, imparts new functions and properties to the whole particle. Nevertheless, this work deserves to be included in the present review because of the synthetic approach employed and the geometry of the structure. The as-synthesized TbPO_4_:Ce^3+^@TbPO_4_:Gd^3+^ nanoparticles were hexagonal prisms with average size of 60 nm, and showed four emission peaks in the visible range, the highest being at 544 nm. The analysis of the dual imaging performance was limited to in vitro studies. The upconverting luminescent imaging and the MR positive contrast enhancement were demonstrated in cellular studies and in agar solutions containing increasing concentrations of nanoparticles, respectively. The longitudinal relaxivity of the Gd-doped nanoparticles was comparable to that of the commercial MRI contrast agent Gd-DTPA.

Other types of upconverting core@shell NHS for multimodal imaging nanoprobes have been reported, mainly consisting of a Gd doped core coated by an upconverting shell or vice versa. In this sense, NaYF4, NaGdF4, NaLuF_4_, and NaYbF_4_ are the most commonly used host materials. To mention some examples, NaYF_4_:Yb/Er/Tm@NaGdF_4_ nanoparticles further coated with a SiO_2_ shell were developed as magnetic/luminescent imaging tools, and as chemo-radiotherapy agents, and are described in detail in the next section [77]. Similarly, NaYF_4_:Yb/Tm/Gd@NaGdF_4_ core@shell nanoparticles functionalized with Angiopep-2 were developed for dual MR and NIR-to-NIR UCL imaging of glioblastoma; Angiopep-2 was employed as it binds to the low density lipoprotein receptor related protein (LRP) [57]. These nanoparticles, whose final hydrodynamic diameter was around 48 nm, could cross the blood–brain barrier (BBB) through ANG-mediated transcytosis both in vitro BBB models and in vivo; they also exhibited superior imaging performances as compared to the clinically used MRI contrast Gd-DTPA and fluorescent dye 5-ALA.

In another study from the group of Yuxin Liu, NaGdF_4_:Yb^3+^,Er^3+^@NaYF_4_ core@shell upconversion nanoparticles were synthesized for a peculiar application that does not fall within the scope of this review but that is worth mentioning. Indeed, the nanoparticles were exploited for detecting the blood distribution of a fluorescent drug, doxorubicin, in vivo. To this aim, the nanoparticles were deposited onto the inner wall of a capillary glass tube connected with the 980 nm laser diode and an optical fiber, and then inserted into the blood vessel of a living mouse. The emission wavelength of the upconverting domain was adsorbed by the circulating doxorubicin, whose fluorescence was then recorded [78].

Interestingly, more complex systems made of multi-shelled upconverting nanoparticles are also developed and tested as multimodal imaging probes. The growth of multiple layers enhances the upconverting luminescence performance, as it reduces the energy losses at the nanoparticle surface; the multiple layers also prevent undesired energy transfer processes between activator/sensitizer ions and enable the combination of multiple functions into a single structure. Wang et al. [58] developed core@shell1@shell2@shell3 NaLuF_4_:Gd/Yb/Er@NaLuF_4_:Yb@NaLuF_4_:Nd/Yb@NaLuF_4_ NPs through subsequent epitaxial growth of the shells over the core. The nanoparticles size shifted from 27.2 ± 0.9 nm in the case of the core up to 42.4 ± 1.1 nm for the three-shelled NPs, as determined by TEM measurements; both the UC and DC luminescence properties were characterized prior to in vivo studies. Upon administration in healthy mice, the signal distribution and enhancement were monitored via NIR UC (under 808 nm laser excitation) and CT imaging, revealing the classic accumulation in liver and spleen within 3 h after injection.

**Figure 4:**
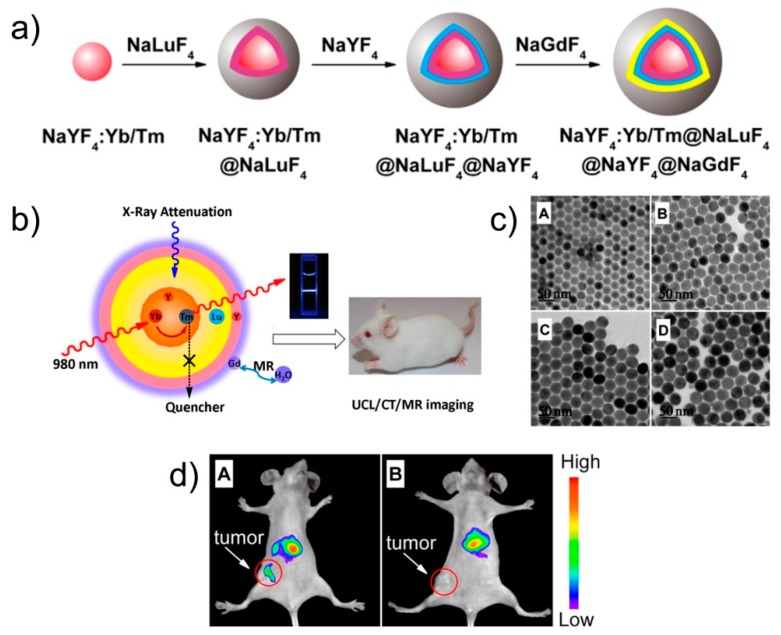
(**a**) Sketch showing the preparation of the multi-shelled NaYF_4_:Yb/Tm@NaLuF_4_@NaYF_4_@NaGdF_4_ NPs. (**b**) Trimodal imaging potential of the nanoparticles. (**c**) TEM images of the nanoparticles at each step of preparation. (**d**) In vivo UCL imaging of Hela tumor-bearing nude mice at 3 h after intravenous injection of nanoparticles with (left) and without (right) folic acid functionalization. Adapted with permission from *Anal. Chem.* **2013**, *85*, 12166−12172 [59]. Copyright 2013 American Chemical Society.

Another example of multi-shelled nanoparticles was provided by the group of Yan, who prepared core@shell1@shell2@shell3 NaYF_4_:Yb/Tm@NaLuF_4_@NaYF_4_@NaGdF_4_ nanoparticles through seeded growth approach and made them water dispersible by replacing oleic acid with polyacrylic acid (Figure 4) [59]. The in vivo trimodal (through UCL, CT and MR) imaging performance was demonstrated upon administration of the nanostructures into normal nude mice via tail vein, and monitoring of the signal distribution into the whole body. As expected, the nanoparticles rapidly accumulated in the liver and in the spleen, becoming clearly distinguishable in these organs at 30, 40 and 120 min post-injection for UCL, MR and CT imaging techniques, respectively. Additionally, the nanostructures were functionalized with folic acid, prior to being injected into nude mice bearing Hela tumors for in vivo UCL imaging of folate receptor positive tumors. The UCL signal was visible at the tumor at 3 h post-injection in the tail vein.

The introduction of a radioactive isotope, such as ^153^Sm^3+^, into the lanthanide nanocrystal may provide an additional function to the nanoparticle, as reported in the work of Sun et al., who prepared core@shell NaLuF_4_:Yb,Tm@NaGdF_4_(^153^Sm) nanoparticles as four-modal probes for tumor angiogenesis imaging [60]. More specifically, UCL, CT, MR, and SPECT imaging was assessed: nude mice bearing KB human oral tumor were intravenously injected with the nanostructures and imaged after 1 h with UCL, X-ray CT, MRI, and SPECT imaging devices. The combination of several imaging techniques allows for a more accurate reconstruction and 3D visualization of tumor growth and the blood vessels development. In this study, SPECT imaging analysis evidenced that around 1.7% of the injected nanoparticles reached the tumor, while CT imaging showed the distribution of the nanoparticles through the rudimental blood tumor circulation. On the other hand, MRI provided a broad overview of the tumor mass and UCL imaging did not produce any distinguishable signal due to low penetration of the wavelength source (980 nm laser with a power density of 50 mW/cm^2^).

One concern regarding these materials is that the excitation conditions needed are quite restricted in the case of UCL imaging; for optical analysis, on the other hand, a critical issue can be the poor penetration. Despite this, however, the data reported here show the encouraging potential and the versatility of this class of NHS.

## 3. Therapy and Theranostics in Nanomedicine

The application of nanoheterostructures in therapy is a rapidly growing hot topic in recent years. It is noteworthy that almost half of the papers cited in this section have been published in last four years. At present, few nanoparticles formulations reach the market for therapeutic application; this indicates how the research on therapeutic heterostructures is a young emerging field.

Thus far, magnetic nanoparticles lead the research on the topic, due to the presence on the market of formulations (e.g., Endorem and Resovist) already employed for MRI technique (see Section 2.1.1). Some clinical trials on the use of magnetic nanoparticles for magnetic hyperthermia treatment of cancer are currently ongoing [79,80]. Regarding the use of nanoparticles for hyperthermia, a critical issue is the minimum concentration of particles needed to observe a significant temperature increase; often, in fact, the intravenous injection of the particles leads to their accumulation at the target site at concentrations not sufficient to cause the required therapeutic temperature (43–45 °C). The most effective way to achieve a significant concentration is the direct intratumoral injection; this protocol, however, has a limited application in clinical studies, as it can only be used for intracellular hyperthermia. At present, research is focused on the design of innovative nanoparticles formulations to overcome this problem; core@shell nanoparticles may provide a significant answer to this, due to their enhanced efficiency.

Next to hyperthermia, PTT is the most commonly used technique in nanoparticle-based cancer therapy. As described in detail in the next section, PTT is based on the easy preparation of several metallic nanostructures, such as gold nanoparticles, and the derived heterostructures. Furthermore, differently from magnetic hyperthermia, to trigger the PTT stimulus, no sophisticated equipment is needed; in fact, this can be done with a simple infrared laser source.

**Scheme 2:**
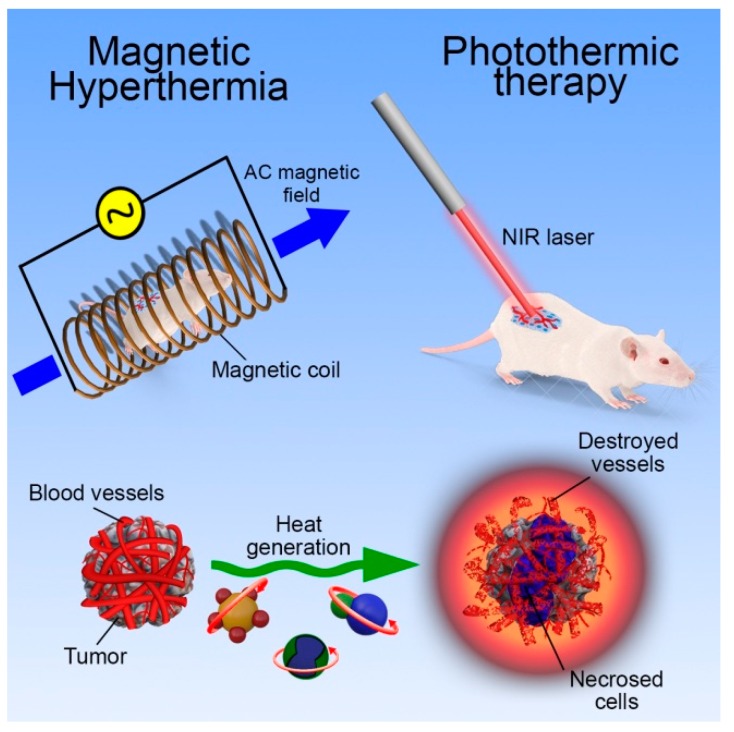
The sketch depicts the most common techniques in nanoparticle-based therapy, magnetic hyperthermia and photothermal therapy. The target is the ablation of malignant cancer cells. To this aim, both techniques exploit the generation of local heat as therapeutic effect, actuated by alternating magnetic field for magnetic hyperthermia and by laser irradiation for PTT, respectively.

As mentioned in the previous sections, one of the advantageous features of NHS is the co-existence of two or more domains in a single system, which can perform different functions at the same time, enabling for instance both the imaging and then the treatment of a tumor mass. The combination of diagnosis and therapy is commonly referred to as “theranostics”. This term generally indicates tools that can be employed for both diagnosis (e.g., MRI, CT, etc.) and therapy (e.g., PTT), and that can also be used as a tool for the delivery of drug or reporter for other techniques (e.g., PET). In the next sections, we report a description of the nanoparticle-based therapeutic techniques (Scheme 2) and the analysis of the theranostic NHS; some examples of nanostructures employed for the eradication of resistant bacterial infection are also discussed. It is worth highlighting that, in this context, the use of nanoparticles reduces the need of antibiotics as bacteria can be killed by applying external stimuli, even in remote body areas.

### 3.1. Therapeutic Techniques

In medicine, and particularly in cancer therapy, heat is considered as a healing tool. Indeed, hyperthermia treatment is based on the well-established evidence that cancer cells are more sensitive to temperatures above 41 °C than healthy cells [81]. Different approaches such as whole body hyperthermia, radiofrequency hyperthermia, and inductive hyperthermia were employed in the past to raise the temperature of tissues. For hyperthermia to have the maximum effect, a crucial point is that only the region affected by the pathology has to be heated, thus avoiding or limiting unspecific hyperthermia treatment of healthy tissues [82].

Considering this, superparamagnetic iron oxide nanoparticles (SPIONs) emerged as optimal candidates, as a spatially limited selective heating can be achieved. Magnetically mediated hyperthermia consists in the localized heating of the area, placing the magnetic nanoparticles in the desired areas and applying an alternated magnetic field (AC). In these conditions, magnetic nanoparticles generate heat by mainly three types of loss processes: hysteresis loss, and Neel and Brown relaxation processes [83]. The relative contribution of each of these processes is heavily affected by the magnetic performance and, hence, by the particle size. The magnetization relaxation is mainly due to the combined effects of the physical rotation of the particle (Brownian relaxation) and the internal rotation of the particle magnetic moment (Neel relaxation) when trying to follow the alternating direction of the magnetic field. Hysteresis loss, on the other hand, is the dominant relaxation mechanism when the nanoparticles are above the superparamagnetic limit, as they become ferromagnetic. The heating ability of magnetic nanoparticles under an alternating magnetic field is expressed by the specific absorption rate (SAR), which takes into account the power loss per magnetic material mass. SAR of a magnetic nanoparticle is strongly related to its structural and magnetic properties.

In addition to the application of a magnetic field, the radiation within the UV-IR spectrum has also been reported as an effective therapeutic trigger; such tool can be used for two different methodologies, namely photothermal and photodynamic therapy (PTT and PDT, respectively). Although both techniques employ electromagnetic radiation, the mechanisms induced by the radiation are different. In PDT, a photosensitive molecule is excited by a specific wavelength to produce reactive oxygen species [84]. The main limit of PDT is that its use is generally restricted to superficial tumors, due to the limited penetration depth of the required radiation. Moreover, ROS production can be achieved only in the presence of oxygen.

In the case of PTT, low energy radiation (between 700 and 1400 nm) is generally employed to treat the diseased cells; radiations with these wavelengths are poorly absorbed by tissues and blood, thus minimizing the damage of the surrounding healthy tissues. With this technique the materials excited by the light release the absorbed energy as heat, thus leading to the local increase in temperature, as it occurs in magnetically mediated hyperthermia. The local heating in turn provokes damage of the surrounding cells up to necrosis of the tumor mass. In the past, organic molecules coordinated to transition metals were applied in PTT, but their poor photostability limited their exploitation in clinical settings [85]. Recently, nanoscaled systems have become the best performing materials in PTT. In addition to their enhanced optical properties, nanomaterials show high permeability in vivo; moreover, their high retention effect in the tumor environment leads to an increased number of particles accumulated at the target site. Nanomaterials used in PTT include metallic nanoparticles (e.g., gold, silver, and copper), semiconductor (e.g., carbon nanotubes), rare earth ion-doped nanocrystals and carbon based and organic nanoparticles [86].

In these three techniques, the use of ultrasmall nanoparticles with long circulation time and high accumulation capacity at the tumor could allow more localized heating of a restricted targeted region, overcoming side effects and limitations of other therapeutic approaches.

### 3.2. Nanoheterostructures for Therapy and Theranostics

At present, the core@shell geometry is the most exploited in NHS designed for therapeutic purposes. In these systems, the two domains can contribute to multiple functions, working as bimodal therapeutic agents or behaving as both imaging and therapeutic tools, as shown in Table 2 that reports the NHS for therapy and theranostics presented soon after. Wang et al. reported the preparation of magnetic structures, consisting of cobalt ferrite/manganese ferrite core@shell [87]. The hydrophobic nanostructure was transferred in water by the polymerization of pyrrole by Fe^3+^ in the presence of polyvinyl alcohol (PVA) and CoFe_2_O_4_@MnFe_2_O_4_ NPs. The nanocomposite was designed to combine simultaneously the photothermal effect of pyrrole and the magnetothermal properties of the inorganic core@shell NPs. The magnetic nanoparticles included in the polymer matrix showed a decrease of the *Ms* and an enlarged hysteresis loop compared to the starting NPs. The composite developed a SAR of 930 W·g^−1^ at 200 kHz, with an applied field of 60.6 kA·m^−1^. Under application of an 808 nm laser together with an alternating field of high frequency, 4T1 cells, a model cancer cell line incubated with nanocomposites was killed. The individual treatments affected the cell viability to 58% and 68% in the cases of photothermal therapy and magnetothermal therapy, respectively. The combination of the two approaches exhibited a synergistic effect. This work is one of the few models of magnetic hyperthermia-based NHS.

The combination of two magnetic materials in the same structure enhances the properties of the whole system [88]. Within this frame, Zhou et al. described the preparation of PEG-coated core@shell nanoparticle based on an iron core and a magnetite thin shell (Fe@Fe_3_O_4_ NPs). The nanoparticles displayed a soft ferromagnetic behavior with a remnant magnetization of 18 emu/g and a coercivity of 160 Oe. Moreover, they had a magnetization value of 113 emu/g at a 6 kOe magnetic field, thus higher than standard magnetite. The NPs showed a triple functionality: magnetic targeting, MR imaging and photothermal therapy. Indeed, although gold nanorods are the standard particles for PTT, in this study, magnetic Fe@Fe_3_O_4_ NPs were excited by NIR radiation and successfully exploited for PTT. The triple functionality of this system was demonstrated in vivo in a hypodermic xenograft tumor. In detail, by applying a permanent magnet on the mouse skin, the NPs were magnetically delivered in proximity of the tumor mass. After 12 h, the NPs accumulated at the tumor, as demonstrated by the enhancement of the MRI signal (threefold) and by the increase in temperature after laser irradiation (at 808 nm, twofold), in comparison with mice treated without magnetic targeting. The accumulation of a considerable amount of NPs at the tumor provoked a strong therapeutic effect by means of PTT-mediated cancer cells ablation [89].

In a very recent study, core@shell Fe@γ-Fe_2_O_3_@H-TiO_2_ nanocomposites were prepared by one-step reduction of Fe and Ti precursors in a hydrogen atmosphere, and tested as theranostic agents in vivo. The nanostructures showed a size around 150 nm, which is close to the recommended threshold for nanoparticles to be applied in biomedical field [90]. Thanks to the presence of the hydrogenated TiO_2_ shell, the nanoparticles have a strong absorption in the NIR region, enabling their use as multimodal photothermal (PT) and photoacoustic PA imaging probes, as well as MR imaging modality mediated by the magnetic core. Once the biocompatibility of the nanostructures coated with PEG in healthy mice after 15-days administration was assessed, in vivo imaging experiments were performed in mice bearing H22 tumors subcutaneously inoculated into the left armpit of the animal. When the tumor size reached about 60 mm^3^, the nanostructures were intravenously injected and PTT imaging was performed using an infrared camera upon exposure of the tumor to 808 nm laser light. Similarly, PA and MR imaging were performed with the suitable setups; this demonstrated the trimodal imaging performance, which were enhanced by the magnetic targeting of the particles at tumor site under the application of a magnetic field. Additionally, the temperature increase mediated by the 808 nm NIR light irradiation (power density at 2 W cm^−2^ for 5 min) and the magnetic field-driven tumor targeting were combined to test the therapeutic potential of Fe@γ-Fe_2_O_3_@H-TiO_2_ nanocomposites: after 14-day treatment, a significant tumor mass reduction was recorded as compared to tumors not exposed to thermal treatment.

Photothermal therapy of cancer is probably the most studied therapeutic application of heterostructures; this is due to the availability of various metallic materials and to the possibility of designing different structural schemes and of synthesizing multiple types of particles [98]. Fan et al. reported the synthesis of multifunctional magnetic/plasmonic core@shell NPs. The aim of the study was to sort the cancer cells by magnetic targeting, and then to destroy them by photothermal radiation. The gold shell was functionalized with Cy3-labeled S6 aptamers for fluorescent detection and specific breast cancer cell recognition. In the experimental setup, three cell populations (namely, SK-BR-3 breast cancer cells, HaCaT keratinocytes, and LNCaP prostate cancer cells) were mixed in the same plate and administered with the NHS: the magnetic separation capacity of breast cancer cells was significant, even at very low percentage of positive cells (0.001%) in the mixed population. Moreover, under a 670 nm light irradiation at 2.5 W/cm^2^ for 10 min, targeted cancer cells were selectively damaged [91]. Recently, Ju et al. synthesized monodisperse Au-Fe_2_C Janus nanoparticles with an overall size of 12 nm. These nanoparticles were demonstrated to be a relevant agent for MRI (r_2_ was 210.6 mM^−1^·s^−1^) and CT, whereas the broad absorption in the near-infrared range guaranteed for a possible use in photothermal hyperthermia (irradiation of the tumor at 808 nm). The combination with an affibody boosted the accumulation of the nanocomposite at the target and resulted in a complete ablation of the tumor, with no evident side effects [92].

**Figure 5:**
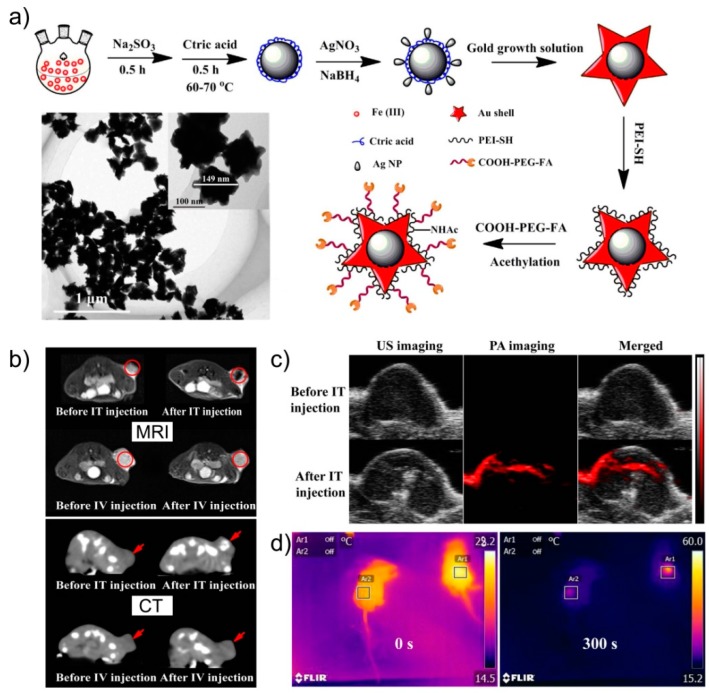
(**a**) Scheme of synthesis of Fe_3_O_4_@Au nanostars and corresponding TEM image. (**b**) In vivo MRI/CT imaging of a xenografted tumor model before and at 0.5 h post intratumor and at 6 h post intravenous injections of Fe_3_O_4_@Au NHS. (**c**) In vivo ultrasound/PA images of tumors before and at 0.5 h post intratumor injection of Fe_3_O_4_@Au nanostars. (**d**) Full-body photothermal images of mice after intratumor injection with 0.1 mL PBS (control, left mouse) or Fe_3_O_4_@Au NSs (right mouse), followed by exposure to an 808 nm laser with a power density of 1.0 W/cm^2^, at a time point of 0 and 5 min. Adapted with permission from *Sci. Rep.* **2016**, *6*, 28325 [93]. Copyright 2016 Nature Publishing Group, licensed under a Creative Commons Attribution 4.0 International License.

Hu et al. synthesized Fe_3_O_4_@Au core@shell nanostars for cancer theranostic through multimode imaging via MR, CT and PA and photothermal therapy (Figure 5) [93]. First, Fe_3_O_4_ NPs were synthesized via a reduction route; this was followed by the formation of Fe_3_O_4_/Ag seed particles that were successively placed in an Au solution, to promote the growth of the star-like gold shell. The magnetic/plasmonic nanostars displayed an average size of 150 nm and were functionalized with carboxy-PEG-folic acid for targeting tumor cells over-expressing folate receptors. Prior to animal studies, the trimodal imaging capability was tested with phantoms and in vitro; then, the nanostars were intravenously or intratumor injected into mice bearing xenograft HeLa tumors and images were collected by MR, CT and PA at 30 min (intratumor) and at 6 h (intravenous) post injection. As expected, the contrast signal was stronger when the nanoparticles were injected directly into the tumor than when systemically injected. Finally, the strong NIR absorption at 808 nm of the gold shell was exploited for the photothermal ablation of HeLa tumors in vivo. After intratumor injection of the nanoparticles, a power density of 1.0 W/cm^2^ was applied at specific time intervals leading to tumor ablation at seven days post-injection without any effects on the mean survival rate of the treated mice.

Tian et al. synthesized ultrasmall (8.5 nm) Fe_3_O_4_@Cu_2-x_S core@shell NP made of a magnetite core and a copper sulfide shell. After deposition of the shell, the initial magnetization value decreased up to 60%, nevertheless the resulting core@shell displayed a good r_2_ value (141 mM^−1^·s^−1^). The NPs were intratumorally injected in a subcutaneous tumor model for in vivo MRI analysis. Thanks to the strong absorption in the near-infrared region, the copper sulfide shell was exploited for in vitro and in vivo photothermal ablation of cancer cells. Ex vivo analysis revealed an increase of necrosis in treated tumors [94]. More recently, Chen et al. reported the preparation of core@shell Pd@Au nanoplates for image-guided photothermal cancer therapy. In detail, hexagonal Pd nanosheets with an average edge length of 15 nm and a thickness of 1.8 nm were synthesized and then used as seeds for the formation of bimetallic nanoplates. The growth of Au on Pd nanosheets was achieved by chemically reduction of AuPPh_3_Cl. The obtained Pd@Au nanoplates were successfully employed as contrast agents by means of photoacoustic and computed tomography imaging. After intravenous injection, the nanoplates accumulated at the tumor site by EPR effect. Moreover, under NIR laser irradiation at 808 nm at a power density of only 0.5 W·cm^−2^, photothermal ablation of tumors was achieved. Notably, this study demonstrated that 2D nanoparticles possess imaging and therapeutic properties comparable to those of spherical or cubic structure; the long-term safety of palladium, however, still needs to be assessed [95].

In a further example of full-metallic structure, branched Au–Ag NPs were prepared by Li et al. through galvanic replacement. In detail, standard citrate-coated Ag seeds were synthesized in aqueous solution followed by the replacement of silver atoms with gold. The use of a mild reductant such as hydroquinone HQ during the branching step avoided the separate nucleation of gold NPs. By varying the ratios of Ag seeds, HAuCl_4_ and HQ, the absorption of the composites was shifted in the near-infrared (NIR) region, thus enabling their use as photothermal agents in cellular studies [96].

Lanthanide based upconverting heterostructures are also emerging as flexible theranostic nanotools. Core@shell nanoparticles are generally prepared by epitaxial growth of one or multiple layers over an upconverting cores: each domain is typically doped with different ions that provide peculiar physical properties to the whole heterostructure. To cite some, NaYF_4_:Yb/Er/Tm@NaGdF_4_ nanoparticles were developed as magnetic/luminescent imaging tools and as chemo-radiotherapy agents in HeLa-tumor-bearing mice [77]. In this case the inorganic core displays upconverting luminescent properties, while the Gd present into the shell provides the magnetic behavior. The nanoparticles were coated with a porous SiO_2_ shell to provide water solubility and possessed a narrow size distribution of 26.6 ± 1.9 nm. In vivo MRI experiments were performed on nude mice bearing xenograft tumor, using a 3.0 T human MRI scanner, while a 0–10 W adjustable 980 nm semiconductor laser was used for upconversion NIR-to-NIR fluorescent imaging in vivo. After intravenous injection, the NHS successfully reached the tumor site by both active/passive targeting: significant accumulation was observed after 30 min via MRI, and after 3 h post-injection via NIR-to-NIR upconversion luminescence (UCL) imaging. The therapeutic activity was exerted by combining radio- and chemotherapy: to this aim, the porous silica shell was loaded with cisplatin, a drug commonly used in clinic. Then, the drug loaded UC nanoparticles were injected into HeLa xenograft tumors in mice, which were then exposed to high-energy X-ray radiation (equal to 8 Gy). The radiotherapy coupled to the delivery of cisplatin resulted very effective in reducing the tumor volume after 16 days of treatment.

More recently, the group of Prasad have reported the synthesis of core@shell NaGdF_4_:Yb,Er@NaGdF_4_:Yb nanoparticles as theranostic anticancer platform for in vivo CT imaging and photothermal ablation, mediated by dopamine [97]. Indeed, the nanoparticles were coated with a silica shell incorporating dopamine that strongly absorbs light under single 980 nm irradiation. In this system, dopamine assists the photothermal effect of the nanoparticles, leading to a temperature increase up to 41.8 °C within 2 min upon NIR irradiation. The photothermal activity of the heterostructures loaded with dopamine was demonstrated analyzing the progressive size reduction of tumor masses grown in nude mice.

As last topic of this review, we present here a brief section that describes a peculiar biomedical application of the NHS as bactericidal agents, due to the release of metallic ions under specific conditions. Chudasama et al. tested superparamagnetic Fe_3_O_4_@Ag core@shell nanostructures as antimicrobial in Gram-negative and -positive bacterial strains; the systems showed an efficiency higher than silver nanoparticles, likely due to their superior colloidal stability in medium [99]. Recently, in two distinct studies, Huo et al. exploited Au@Ag core@shell NPs in antimicrobial treatment. While the gold domain allows X-ray attenuation in CT imaging, the outer silver shell enables a long-term antibacterial effects [100]. The composite was then functionalized with a monoclonal antibody against methicillin-resistant *Staphylococcus aureus* (MRSA) for selective binding of the pathogen and consequent treatment of related pneumonia [101].

An interesting metallic nanostructure was described by Hu et al.: rod-shaped Au@Ag@Au core@shell@shell nanoparticles were activated as bactericidal agents under NIR excitation. This bimetallic structure was prepared through the synthesis of gold nanorods, followed by a two-step deposition of silver and gold layers [102]. The aim of the study was to control the release of silver ions and the photothermal activity of gold nanorods under NIR laser irradiation, to tailor the bactericidal effect. Indeed, first the outer Au shell melted, resulting in the exposure of the Ag shell; this was followed by the release of Ag^+^. Finally, the photothermal activity of gold nanorods was initiated, leading to 100% killing rate of *E. coli* O157:H7 cells under 20 min NIR irradiation [103].

## 4. Pharmacokinetics of the Nanoheterostructures

Regardless of the physiochemical properties of the materials and of the type of biomedical applications, nanoparticles designed for in vivo use need to interact with the host organism at different levels (i.e., circulating plasma proteins and biomolecules, cells from the immune systems, filtering organs and biological barriers) prior to reach the desired site. This continuous inter-play regulates the pharmacokinetics of the nanomaterials as well as their effects into the host system, in terms of efficacy for the designed scope (both diagnostic and therapeutic) or adverse systemic toxicity, respectively. In the last years, the study of these interactions and of their effects have led to a better evaluation of the clinical relevance and potential of the nano-based systems [104]. It is worth mentioning that the pharmacokinetic of the nanoparticles, i.e., their absorption, distribution, metabolism, and excretion (ADME) profiles, directly depends on the administration route and on the envisaged use on the one hand, and on the morpho-structural features of the materials, on the other hand. In this sense, the “size” matters: indeed, it is crucial that the particles possess dimensions below 200 nm and a uniform size distribution; in fact, particles with larger size, when injected in vivo, may lead to undesired effects, such as thrombus, occlusions and kidney blocks. In addition, it has been reported that nanoparticles with size larger than 200 nm are rapidly coated by plasma proteins and have a short plasma half-life [105].

Other critical features are the composition of the material, the particle charge, the hydrophilicity/hydrophobicity ratio and the surface chemical groups that influence the interactions with the immune system, the circulation time in addition to the safety of the nanoparticles. For instance, adsorption of opsonins onto the nanoparticles surface that occurs through various types of chemical bonds (such as electrostatic, hydrophilic/hydrophobic interactions and van der Waals) determines their biodistribution, because the opsonized nanoparticles are recognized and sequestered by the macrophages of the reticuloendothelial system (RES). Major organs involved in this process are liver and spleen. Thus, it is not surprising that most nanoparticles accumulate into them, leading to a drastic reduction of the amount of circulating particles that can reach the target site [106].

In addition, the administration method of the nanoparticles, such as oral intake, inhalation, subcutaneous, or most commonly injection, may determine their intra-body distribution and fate, as many physiological barriers, such epithelia and vascular endothelium, slow down or block the nanoparticle diffusion and transport [107]. These are just some factors among many others that dictate the ADME profiles of biomedical nanoparticles and have been also considered in the case of NHS. Indeed, many studies herein presented aimed to evaluate the pharmacokinetics of inorganic NHS in addition to study their diagnostic and therapeutic potential in cancer.

Generally, xenograft tumors are grown on rodent models and, when the nanoparticles are administered, two different injection approaches may be chosen: intratumor or systemically. Clearly, major concerns arise in the case of systemic administration, because, following injection, the NHS circulating into the blood stream have to reach the target tissue/site without being altered or blocked. Thus, it is not surprising that the best performances in terms of imaging or therapeutic efficacy are recorded when the nanoparticles are directly injected into the tumor. Reasonably, the biodistribution of the NHS is also affected by the administration route, resulting with the nanoparticles injected into the blood stream those having higher accumulation in RES organs than those injected intratumorally.

In addition, the safety evaluation of the NHS is the most commonly presented analysis: in vivo toxicity and inflammation in major organs, such as heart, liver, spleen, lung and kidney, are performed through histological analysis and blood biochemistry assays, revealing a good compatibility of the materials developed within the time window considered (from 1 to 30 days). In vivo assessment of the NHS toxicity is crucial to predict their metabolization and undesired effects in tissues or organs. Thus, they should always be performed prior to investigating the biomedical potential of the developed materials.

On the other hand, one of the missing analyzed points that should deserve major attention is an accurate study of the in vivo fate (bioaccumulation/excretion) of the NHS. Indeed, most NHS are composed of metal elements, whose slow leakage from the inorganic core and accumulation would generate acute toxic responses upon long time exposure. These are very critical features that can impact the long-term applicability of these materials, as evidenced by the recent reports about Gd-based contrast agents that after years of “safe” use in humans, are now under revision for their long term brain toxicity [108,109].

## 5. Discussion

Multimodal imaging of cancer is essential for the detection and the accurate localization of tumor masses, particularly at an early stage. In addition, combined therapeutic approaches are highly desirable for effective treatments, to reduce the amount of drugs physiologically needed, preventing chemoresistance and unpleasant side effects, and thus improving the overall therapeutic efficacy. Furthermore, when a probe acts both as a diagnostic and therapeutic tool, the follow up of the therapy is easily achievable.

It is therefore not surprising that, thanks to the advances of colloidal chemistry, many new nanostructures displaying multiple properties have been designed and employed as imaging/therapeutic probes, even in animal models. These important results were made possible by the knowledge of the peculiar physical properties of nanosized materials. An example is the possibility to exploit magnetic nanoparticles as imaging tools in MRI and, at the same time, as therapeutic agents in magnetic hyperthermia or therapeutic enhancer, thanks to the magnetic delivery of specific drugs.

As already reported in the Introduction, there are some issues preventing the widespread use of NHS in biomedicine (stability of the colloids, scalability of the synthesis, etc.). In addition, another possible obstacle for the clinical use of NHS systems is related to their size, which is a crucial parameter in nanobiomedicine.

Because of this, the development of innovative NHS, with suitable dimensions and functional properties, is an important step for NHS to be employed, as their performance is in fact comparable to that of other multifunctional systems such as organic templates loaded with different imaging tools and therapeutic molecules. These systems, however, show much larger size (i.e., micrometer scale) and are therefore less suitable for in vivo application.

Thus far, inorganic NHS have been primarily applied as imaging agents, due to their confirmed performance in several imaging techniques, including MRI, CT and optical imaging. Indeed, NHS can improve the diagnostic outcome in terms of both resolution and accuracy, by either enhancing the signal intensity or combining two imaging approaches.

In addition, benefits arising from the heterostructures include the possibility to perform a combined therapy. Therapeutic approaches involving individual nanomaterials have been rarely decisive: in the case of magnetic hyperthermia, for instance, the complete ablation of tumor mass is rarely achieved, probably because of the low nanoparticles concentration at target site, and the poor particle efficiency once internalized in cells [110]. The photothermal therapy, on the other hand, is limited as it can cause significant damage to the surrounding healthy cells under irradiation. Moreover, for both techniques, the generated heat can be dissipated very quickly through floating body liquids; this can lead to a significant decrease in the efficacy of the treatment. A combined approach, instead, has shown to be more effective; for instance, the use of a dual treatments based on single NPs or of a combined organic/inorganic NP-based treatment can lead to effective ablation. Indeed, literature reports that the combination of magnetic hyperthermia with photodynamic therapy [111] or photothermal therapy [112] promoted the total ablation of solid tumors in animals. In a similar approach, the group of Zhao [87] proposed the combination of magnetic hyperthermia and PTT exploiting the presence of iron oxide and gold in the same NHS.

The other main possible problem related to the biological exploitation of the NHS is their cellular and systemic toxicity. Thus far, many studies investigated the biological impact of inorganic nanomaterials, considering several physiochemical factors, including the size, the shape, the composition, the surface chemistry and the charge [113]. Despite these efforts, the molecular mechanisms and the cellular effects of nanoparticle-mediated toxicity are only partially understood, due to the broad variability of nanomaterials developed and the different factors to be considered. The latter also include chemical and physical environmental conditions, such as pH, temperature, ionic strength, proteins adsorption, and the model systems to be used.

A common mechanism of NP-induced toxicity is the production of ROS resulting in the subsequent oxidative stress [114]. As an example, the degradation of iron-based NPs looks to catalyze ROS generation and formation of OH radicals from H_2_O_2_ [11]. Other nanoparticles can induce ROS production under specific biological conditions or by altering organelle homeostasis; an example can be the alteration of mitochondria. In this case, in fact, cellular oxidative metabolism occurs, and the dysfunction may lead to the formation of superoxide anion radicals and of other oxygen-based radicals. High ROS levels generally lead to significant cellular damage by peroxidizing lipids, altering the structure and function of proteins and DNA. This, in turn, results in the activation of the inflammatory responses through the transcription of various pro-inflammatory genes, including tumor necrosis factor-α and IL (interleukin)-1, IL-6 and IL-8. The activation of these molecular and cellular mechanisms is followed by severe cellular genotoxicity, finally leading to cell death [10,115].

In vivo oxidative stress is generally associated with the development of cancer and many degenerative diseases, further than to the alteration of the physiological function of cardiovascular, pulmonary and renal systems [116]. Zhu analyzed and compared the toxicity of three metal oxides nanoparticles, CuO, CdO, and TiO_2_. CuO and CdO NP showed high cytotoxicity and DNA damage, leading to 8-hydroxy-20-deoxysuanosine (8-OHdG) formation, whereas TiO_2_ NP induced up-regulation of the transcription of various pro-inflammatory genes [117]. In the case of gold and silver nanoparticles, many studies confirm that Ag is much more toxic than Au, and its deleterious effects are induced by ROS generation [118,119]. In the case of semiconductor QDs, the release of heavy metal ions from the breakdown of QDs is generally considered the main cause of their toxicity, together with the production of free radicals [120,121].

Regarding the toxicity of upconverting nanoparticles, at present, most studies conclude that they show low or negligible toxicity, both in vitro and in vivo; it has to be considered, however, that this is a relatively young field and more critical investigations need to be undertaken, in particular on the long-term effects of UP NP in animal models [122].

An interesting review has been published, which analyses the role of autophagy and lysosomal alterations as mechanisms of nanomaterial toxicity [123]. It is known that nanoparticles generally entry cells through an endocytosis mechanism and that they are accumulated into the endolysosomal compartments. Subsequent permeabilization of lysosome membrane can occur through several mechanisms, including lysosomal oxidative stress, release of cathepsins and other lysosomal hydrolases, thus leading to lysosomal dysfunction [124]. On the other hand, it has been proposed that nanoparticles, once internalized into cell environment, may undergo autophagic sequestration, whose complex pathway and related dysfunctions are notably associated to cell damage and death.

Finally, we would like to remark that, in the case of NHS, exhaustive investigation of their potential toxic effects deserves more attention and needs powerful experimental evidence both in vitro and in vivo. Therefore, beyond their imaging/therapeutic potential, a primary concern should be to assess thoroughly the toxicological effects of these nanostructures, by adopting common practices and protocols to standardize the operational modes and to simplify cross-analysis of multiple outcomes.

## 6. Conclusions

This review summarizes the recent research results about the development of inorganic nanoheterostructures for application in biomedicine; more specifically, applications in the fields of diagnostics and imaging are considered, including tests and in vivo experiments.

The literature reported confirms the potential of NHS, especially in cancer diagnosis and therapy; in fact, the main advantage of these systems is that it is possible to obtain NHS with different functional properties, by combining different nano-domains. These NHS are therefore multifunctional and can be used for contemporary diagnosis and therapy. The main drawback, on the other hand, is the possible toxicity of these systems; indeed, more studies are needed on this part, to have a clearer picture on the effective risks associated to their use, as well as the achievement of the suitable properties in NHS smaller than 200 nm.

Overall, all research performed on this topic confirms the interest of the scientific community to exploit the properties of these systems and to make them effective tools in various sectors of biomedicine.

## Figures and Tables

**Table 1:** Summary of nanoheterostructures applied in diagnostics.

Heterostructure (Materials)	In Vivo Imaging Technique						Type	Average TEM Size (nm)	DLS/ Z-potential	Surface Coating	Targeting Moiety	Animal Model	Ref.
	MR	CT	PA	PL em.	SPECT	PET							
***Magnetic/metallic nanoheterostructures***													
Fe_3_O_4_@Au	**x**						Core/Shell	12	-/0.49 ± 0.12 mV	PEG	-	Mice bearing a subcutaneous C26 colon cancer	[38]
Fe_3_O_4_@TaO_x_	**x**	**x**					Core/Shell	10	21nm/-	RITC- functionalized silane and PEG-silane	-	Rats bearing xenograft tumors	[39,40]
Fe_3_O_4_@Gd_2_O_3_	**x**						Core/Shell	9	-	Dopamine	-	Sprague Dawley rats	[41]
Fe_5_C_2_@Fe_3_O_4_	**x**						Core/Shell	23	35nm/-	DSPE-PEG-COOH	c(RGDyK) peptide	U87MG tumor bearing mice	[42]
Au-Fe_3_O_4_ labelled with [^64^Cu]	**x**					**x**	Heterodimers	17	24.4 ± 2 nm/ −28.6 ± 1.8mV	LPA-mPEG-2000/ LPA-PEG-2000-NOTA	Anti-EGFR affibody protein	Mice bearing EGFR positive A431 tumors	[43]
Fe_3_O_4_-Ag^125^I					**x**		Heterodimers	23	-	mPEG-LA	-	Kunming mice	[44]
Au-Fe_3_O_4_	**x**	**x**					Heterodimers	25	-	TMAOH	-	Rabbits	[45]
Fe_3_O_4_@Au	**x**						Core/Shell	30	121 ± 5 nm/ −19.1 ± 3.3 mV	DSPE-PEG-COOH	-	4T1-GFP bearing mice	[46]
Au-Pt-IONP	**x**	**x**				**x**	Heterotrimer	25	26.8 ± 0.9 nm/ –16.4 ± 1.2 mV	dopamine terminated PEG_5k_	-	HT-29 tumor bearing mice	[47]
Au@Fe_3_O_4_	**x**	**x**					Flower-like	25	30.4 ± 8 nm/-	Amphiphilic polymer-PEG	-	hepatoma-bearing mice	[48]
Au@Fe_3_O_4_							Flower-like	20	-	TDOPA/MMP/NIRF dye Cy5.5 and PEG5000-SH	-	Mice bearing a SCC-7 tumor	[49]
***Luminescent nanoheterostructures***													
[^64^Cu] doped CdSe/ZnS QDs				**x**		**x**	Core/shell	6	18.5 ± 0.9 nm/-	Amine-PEG-thiol	-	U87MG glioblastoma bearing mice	[50]
[^64^Cu] doped CuIn@ZnS QDs				**x**		**x**	Core/shell	6	23 nm/-	Methoxy-PEG-Thiol	GSH	U87MG glioblastoma bearing mice	[51]
NaYF_4_:Yb^3+^,Tm^3+^@Fe_3_O_4_	**x**			**x**			Core/shell	30	-	Dopamine	-	Lymphatic system of nude mice	[52]
Fe_3_O_4_@NaLuF_4_	**x**	**x**		**x**			Core/shell	300	-	-	-	Nude mice bearing xenograft (HeLa cells) tumors	[53]
Fe_3_O_4_-NaYF_4_@TiO_2_				**x**			Core/shell	250	-/−35.4 mV	hyaluronic acid	-	Nude mice bearing xenograft (S180 cells) tumors	[54]
NaYF_4_:Yb,Er/NaGdF_4_	**x**			**x**			Core/shell	42	62.6 nm/-	PEG-phospholipids	-	Nude mice bearing a U87MG tumor	[55]
Fe_3_O_4_@NaYF_4_:Yb/Er	**x**			**x**			Core/shell	50	138 nm/ +35.1 mV	3-APTES and PEG	Folic acid	Nude mice bearing xenograft MCF7-derived tumor	[56]
NaYF_4_:Yb/Tm/Gd@NaGdF_4_	**x**			**x**			Core/Shell	19	47.9 ± 2.2 nm/ 30.8 ± 0.5 mV	NH_2_-PEG_5k_-SH	Angiopep-2	human breast adenocarcinoma tumor bearing mice	[57]
NaLuF_4_:Gd/Yb/Er@NaLuF_4_:Yb@NaLuF_4_:Nd/Yb@NaLuF_4_		**x**		**x**			Core/Shell	42	-	PEG-conjugated phospholipid	-	Kunming mice	[58]
NaYF_4_:Yb/Tm@NaLuF_4_ @NaYF_4_@NaGdF_4_	**x**	**x**		**x**			Core/Shell	38	55 nm/ −21 mV	Polyacrylic acid	Folic acid	Nude mice bearing Hela tumors	[59]
NaLuF_4_:Yb,Tm@NaGdF_4_(^153^Sm)	**x**	**x**		**x**	**x**		Core/Shell	21	23 nm/-	Citric acid	-	KB tumor grafted in nude mice	[60]

**Table 2:** Summary of nanoheterostructures applied in therapy and/or theranostics.

Heterostructure (Materials)	Type	Imaging Technique				Therapeutic Activity			Average TEM Size (nm)	DLS/Z-potential	Surface Coating	Targeting Moiety	Animal Model	Ref.
		MR	CT	PA	UCL	MH	PTT	DD						
CoFe_2_O_4_@MnFe_2_O_4_	Core/shell					**x**	**x**		70	110 nm/-	PPy/PVA	-	-	[87]
FePt@Fe_2_O_3_	Core/shell	**x**						**x**	7	51 nm/-	PEG	Folic Acid	Balb/c mice, 4T1 tumor	[88]
Fe@Fe_3_O_4_	Core/shell	**x**					**x**		13.4	103 ± 1.1 nm/-	PEG	-	Hypodermic xenograft HeLa tumor	[89]
Fe@y-Fe_2_O_3_@H-TiO_2_	Core/shell	**x**		**x**			**x**		300	-/−8 mV	PEG	-	Mice bearing H22 tumors	[90]
Fe_3_O_4_@Au	Core/shell						**x**		40	55 ± 3 nm/-	PEG	Cy3-modified S6 aptamer	-	[91]
Au@Fe_2_C Janus NPs	Dumbell	**x**	**x**	**x**			**x**		12	33.83 nm/-	PEG	Z_HER2:342_	Nude mice bearing the MDA-MB-231 tumor	[92]
Fe_3_O_4_@Au Nanostar	Core/Shell	**x**	**x**	**x**			**x**		150	224.2 ± 4.9 nm/+14.4 ± 0.2mV	Carboxy-PEG	Folic acid	Mice bearing xenograft HeLa tumors	[93]
Fe_3_O_4_@Cu_2−x_S	Core/Shell	**x**					**x**		8.5	26 nm/-	Polymaleic anhydride	-	Mice bearing xenograft HeLa tumors	[94]
Pd@Au Nanoplates	Core/Shell		**x**	**x**			**x**		30	60 nm/−5 mV	PEG	-	Balb/c mice bearing 4T1	[95]
Au@Ag	Core/Shell						**x**		135	-	Polydopamine	-		[96]
NaYF_4_:Yb/Er/Tm@NaGdF_4_	Core/Shell	**x**			**x**			**x**	45	79.1 nm/-	Silica	-	Nude mice bearing xenograft HeLa tumor	[77]
NaGdF_4_:Yb/Er@NaGdF_4_:Yb	Core/Shell		**x**		**x**		**x**		35	-	Silica/Dopamine	-	Balb/c mice with subcutaneous U14 tumor	[97]

## Abbreviations

AC	Alternated magnetic field
ADME	Absorption, distribution, metabolism, and excretion
CT	Computed Tomography
FDA	Food and Drug Administration
FITC	Fluorescein IsoThioCyanate
IONP	Iron Oxide NanoParticle
IL	Interleukin
LRP	Lipoprotein Receptor related Protein
mAb	Monoclonal AntiBody
MRI	Magnetic Resonance Imaging
NHS	Nanoheterostructures
NIR	Near-InfraRed
NP	NanoParticle
QD	Quantum Dot
PA	PhotoAcoustic
PDT	PhotoDynamic Therapy
PEG	Poly(Ethylene Glycol)
PET	Positron Emission Tomography
PT	PhotoThermal
PTT	PhotoThermal Therapy
ROS	Reactive Oxygen Species
SAR	Specific Absorption Rate
SPECT	Single-Photon Emission Computed Tomography
UC	UpConversion
UCL	UpConversion Luminescence
UV	UltraViolet
8-OHdG	8-hydroxy-20-deoxysuanosine
